# Integrated Structural and Glycoproteomic Profiling Reveals Protein Conformational Remodeling and Biomarkers Across Alzheimer’s Disease Progression

**DOI:** 10.1021/acscentsci.5c02048

**Published:** 2026-01-02

**Authors:** Haiyan Lu, Ching-Yuan Yang, Hua Zhang, Xudong Shi, Penghsuan Huang, Peng-Kai Liu, Zicong Wang, Sanjay Asthana, Cynthia Carlsson, Ozioma Okonkwo, Lingjun Li

**Affiliations:** † School of Pharmacy, 5228 University of Wisconsin-Madison, Madison, Wisconsin 53705, United States; ‡ Biophysics Graduate Program, 5228 University of Wisconsin-Madison, Madison, Wisconsin 53706, United States; § School of Medicine and Public Health, 5228 University of Wisconsin-Madison, Madison, Wisconsin 53792, United States; ∥ Department of Chemistry, 5228 University of Wisconsin-Madison, Madison, Wisconsin 53706, United States; ⊥ School of Medicine and Public Health, University of Wisconsin, Madison, Wisconsin 53705, United States; # Lachman Institute for Pharmaceutical Development, School of Pharmacy, 5228 University of Wisconsin-Madison, Madison, Wisconsin 53705, United States; ∇ Wisconsin Center for NanoBioSystems, School of Pharmacy, 5228 University of Wisconsin-Madison, Madison, Wisconsin 53705, United States

## Abstract

Alzheimer’s disease (AD) is characterized by progressive neurodegeneration and protein misfolding, yet the structural dynamics of proteins and their post-translational modifications during disease progression remain poorly understood. Here, we present an integrated structural and glycoproteomic analysis of paired serum and cerebrospinal fluid (CSF) samples from individuals across three clinical stages: normal cognition, mild cognitive impairment, and AD. Using limited proteolysis mass spectrometry (LiP-MS) combined with high-field asymmetric waveform ion mobility spectrometry and data-independent acquisition, we identified 54 proteins exhibiting structural alterations, two of which (clusterin and ceruloplasmin) showed structural changes in both serum and CSF. Furthermore, our findings reveal potential crosstalk between protein structural changes and N-glycosylation, supported by correlations between LiP-derived structural features and glycosylation patterns in key proteins, such as haptoglobin and kininogen-1. This study demonstrates that integrating structural proteomics with glycoproteomics in matched serum and CSF samples enhances biomarker discovery and provides novel insights into the molecular mechanisms of AD. Our approach offers a powerful platform for identifying robust, minimally invasive biomarkers and for understanding post-translational modification-induced protein remodeling in neurodegenerative diseases.

## Graphical abstract

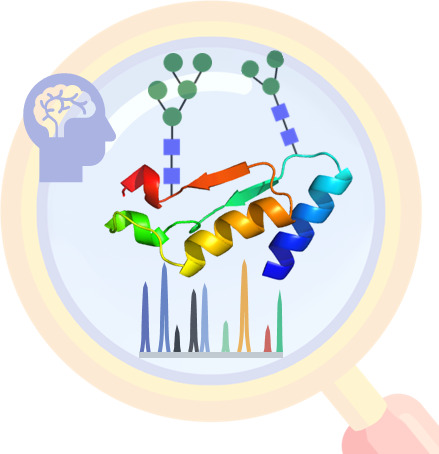

## Introduction

Alzheimer’s disease (AD) is a progressive and debilitating neurodegenerative disorder with rising prevalence in aging populations.^1^ Its long preclinical phase, which can span 20 to 30 years, poses significant challenges for early detection and diagnosis.^2^ Consequently, there is an urgent need for sensitive biomarkers that can facilitate early diagnosis, monitor disease progression, and guide the development of effective therapeutic strategies.^3,4^ Cerebrospinal fluid (CSF), serum, plasma, and tissue are valuable clinical specimens for AD biomarker discovery.^5^ Among analytical platforms, mass spectrometry (MS) stands out for its high sensitivity, throughput, and molecular specificity.^6^ Recent MS-based studies have focused on identifying potential AD biomarkers by employing proteomics to examine changes in protein abundance^7–11^ or N-glycoproteomics to investigate aberrations in N-glycopeptides, N-glycoproteins, and N-glycosites^12,13^ in individual CSF, serum, plasma, and tissue samples. However, global proteomic and N-glycoproteomic analyses often overlook critical information regarding structural changes, and few studies have investigated how N-glycosylation influences protein structural dynamics.

AD pathology is closely linked to protein misfolding and conformational alterations.^14^ Structural alterations in amyloid β and Tau proteins are central to disease progression and severity.^15–17^ Given this association, a global analysis of the human structural proteome may uncover novel structural biomarkers that reflect dynamic conformational changes, provide direct insights into protein activity, and reveal functional roles during disease progression.^18^ These structural biomarkers are invaluable for elucidating molecular mechanisms and identifying novel therapeutic targets in AD. In addition to protein structural abnormalities, glycosylation plays a significant role in AD pathogenesis.^13^ Previous studies have revealed aberrant glycosylation in several key AD-related proteins, such as amyloid-beta precursor protein, Tau, transferrin, Reelin, and collapsin response mediator protein 2.^19^ Since glycosylation can modulate protein structure,^20^ understanding its impact on conformational dynamics is essential for advancing our knowledge of AD mechanisms.

Limited proteolysis mass spectrometry (LiP-MS) is a powerful technique for detecting protein structural changes in complex biological samples.^21,22^ Our previous studies revealed protein structural changes in human AD CSF samples using label-free quantitation with LiP-MS technology.^18^ Additionally, high-throughput quantitative analysis of protein structural changes in human AD serum samples was enabled through an innovative combination of *N,N*-dimethyl leucine isobaric labeling and LiP-MS.^23^ These studies confirmed that LiP-MS is effective for profiling protein structural changes in complex AD serum and CSF samples. However, these studies did not explore the potential relationship between structural changes in proteins from paired serum and CSF samples, nor did they investigate the correlation between protein structural changes and N-glycosylation. Moreover, matched biomarkers in serum and CSF for AD are significantly more reliable, yet they are currently unavailable. The discovery of consistent biomarkers in matched CSF and serum samples would improve compliance with early diagnostic screening in clinical settings, due to the potential implementation of less invasive serum tests compared to more invasive CSF-based diagnostics.

To address these critical gaps, we performed structural proteomics on paired serum and CSF samples using LiP-MS, integrating high-field asymmetric waveform ion mobility spectrometry with data-independent acquisition to enhance the depth and coverage of the AD proteome. Furthermore, we conducted N-glycoproteomics via hydrophilic interaction liquid chromatography enrichment, followed by site-specific mapping of N-glycosylated peptides. Leveraging these technological advancements, we aim to assess the consistency of protein structural changes between paired serum and CSF samples, elucidate the potential relationship between protein structural changes and N-glycosylation, and identify key biomarkers to support the development of improved diagnostic and therapeutic strategies for AD.

## Results

### Study Design and Condition Optimization

CSF and serum are valuable clinical specimens for AD research, as they contain measurable protein biomarkers that reflect physiological and pathological changes associated with the disease.^11,24^ CSF, due to its direct contact with the brain, is particularly well-suited for detecting brain-related alterations,^25^ while serum offers the advantage of easier accessibility. To identify matched biomarkers in both serum and CSF for early diagnosis and monitoring of AD progression, this study utilizes paired human serum and CSF samples across three clinical stages: cognitively normal individuals (Normal), mild cognitive impairment (MCI), and AD (see Table S1 for details).

Given the high complexity and broad dynamic range of protein abundances in serum and CSF, detecting low-abundance proteins remains a significant challenge. Pooled serum samples from three groups—AD, MCI and Normal—were used to evaluate whether Top14 abundant protein depletion columns enhance proteome depth in serum analyzed by LiP-MS. As shown in multiple plots (Figure S1), Pearson correlation coefficients exceeded 0.98 at the protein level and 0.85 at the peptide level, indicating high reproducibility of the depletion approach. Pairwise comparisons indicated that, in the LiP-MS group, a greater number of conformotypic peptides (Figure S2) and potential structural variants (Figure S3) were identified in depleted samples compared to nondepleted counterparts during AD vs MCI and AD vs Normal comparisons. Furthermore, analysis of Trypsin/LysC-only digested samples showed consistently higher protein identifications in depleted samples across AD, MCI and Normal groups (Figure S4). Collectively, these results suggest that depletion of the Top 14 high-abundance proteins enhances overall protein detection. To assess potential protein loss during depletion, flow-through fractions from AD, MCI and Normal serum samples were analyzed. Venn diagrams (Figure S5) revealed that within each group, one sample exhibited a distinct protein profile compared to the other two, and a subset of proteins was consistently lost across all three samples, reflecting systematic losses associated with the depletion procedure.

Although depletion of the Top14 abundant proteins enhances overall proteome coverage, it may introduce experimental variability and lead to the loss of potentially relevant AD-associated proteins. To address these limitations, we next evaluated advanced MS acquisition strategies to improve detection sensitivity and reproducibility. Recent evidence highlights the benefits of data-independent acquisition (DIA) in discovery proteomics due to its unbiased selection of peptide precursors,^26^ while high-field asymmetric waveform ion mobility spectrometry (FAIMS) enhances protein identification by separating ion groups using compensation voltages (CVs).^27^ Using serum as a model, our results demonstrate that FAIMS-DIA substantially improves both the coverage and reproducibility of the AD serum proteome (Figure S6). Building on the enhanced depth and reproducibility achieved with FAIMS-DIA, we next explored strategies to maximize proteome coverage and enable reliable identification of structural features. In the context of LiP-MS within a DIA workflow, the presence of half-tryptic peptides necessitates a study-specific library.^21,28^ To this end, we compared library-based and library-free analysis methods using spectra generated from offline HpH fractionation samples acquired via data-dependent acquisition (DDA) and sequence libraries, respectively. Overall, our results indicated that DIA library-based analysis resulted in more protein identifications and peptide identifications compared to the library-free method (Figure S7).

An overview of the study workflow is outlined in Figure 1. In the structural proteomics analysis, we established a robust library for structural feature identification by analyzing fractionated pooled samples using DDA integrated with FAIMS (LiP-FAIMS-DDA-MS). To generate the library, CSF and serum samples were pooled into three conditions (AD, MCI, and Normal), including LiP-treated and the Trypsin/LysC-only preparations. For individual sample analysis, we employed LiP-MS technology combining FAIMS with DIA (LiP-FAIMS-DIA-MS). In the LiP-MS group, extracted proteins were first subjected to limited proteolysis under native conditions using proteinase K to generate LiP samples. Subsequently, these LiP samples, along with Trypsin/LysC-only samples, were then digested under denaturing conditions using Trypsin/LysC. The resulting digests were analyzed by liquid chromatography-tandem mass spectrometry (LC-MS/MS) with FAIMS. In parallel, N-glycoproteomics was performed by enriching glycosylated tryptic peptides via hydrophilic interaction liquid chromatography (HILIC) enrichment, followed by LC-MS/MS data acquisition in DDA mode without FAIMS. Together, this study aims to identify matched AD biomarkers in both serum and CSF and to explore the potential correlation between protein structural changes and N-glycosylation through integrative analysis.

**Figure 1:**
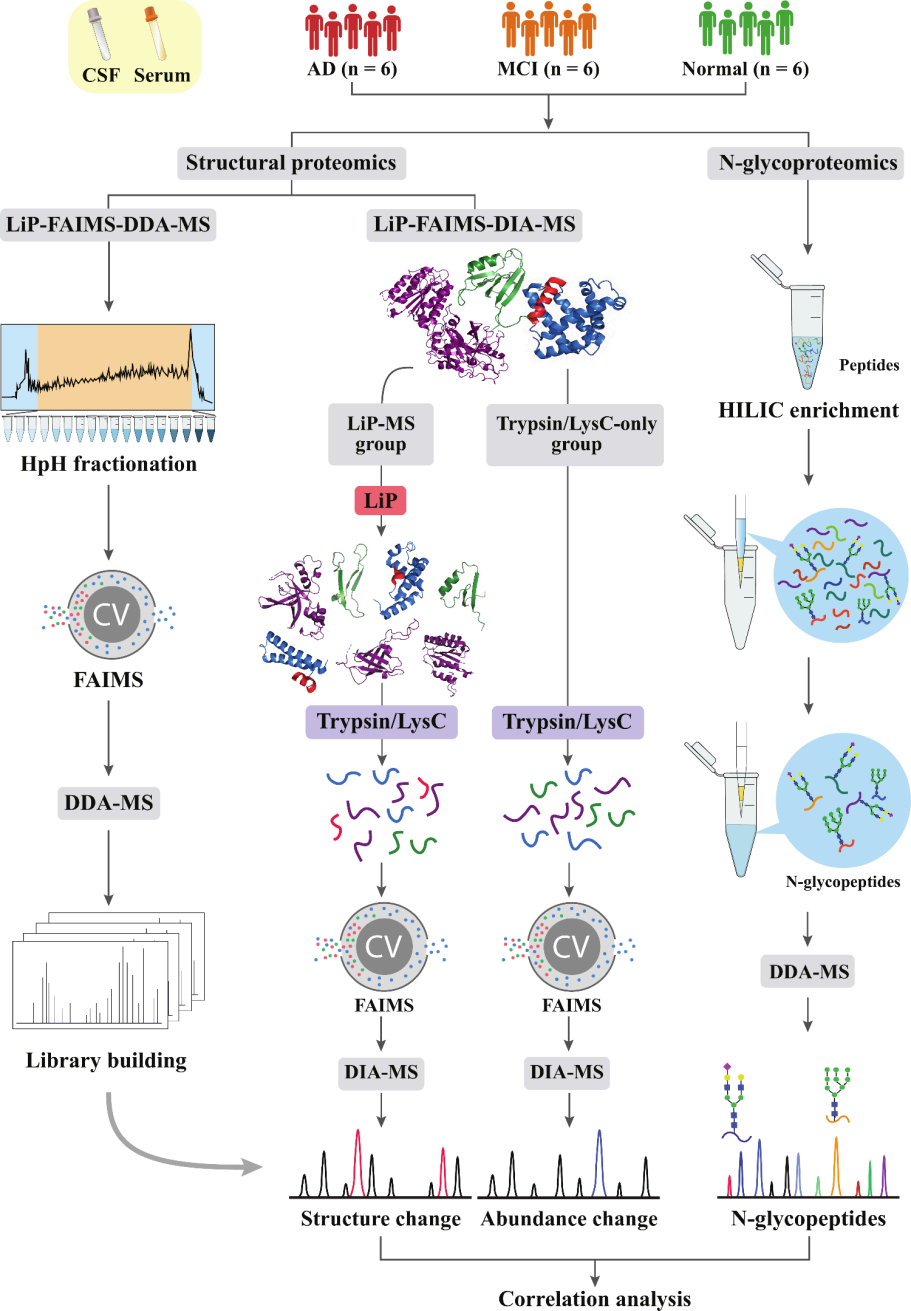
Schematic overview of the study design. This study includes paired serum and CSF samples from three clinical stages: Normal, MCI and AD patients (*n* = 6 per group). In the LiP-FAIMS-DDA-MS workflow, fractionated pooled samples were used to generate a spectral library under DDA mode for identification of DIA data. The LiP-FAIMS-DIA-MS technology was utilized to acquire structural proteomics data from individual samples. N-Glycoproteomics was enabled by HILIC enrichment, and data were acquired under DDA mode without FAIMS.

### Global Identification of Protein Structural Changes in Serum and CSF

To comprehensively investigate structural biomarkers associated with AD, we employed a library-based LiP-FAIMS-DIA-MS workflow to perform an unbiased analysis of protein structure and abundance changes in paired serum and CSF samples from individuals across three clinical stages: AD, MCI, and Normal. In the Trypsin/LysC-only group, we identified over 470 proteins in serum and more than 1100 proteins in CSF (Figure S8a). In the LiP-MS group, approximately 20,000 peptides were detected in both biofluids (Figure S8b). After normalizing for protein abundance changes across conditions, significantly altered LiP peptides — termed “conformotypic peptides” — were used to pinpoint specific protein regions undergoing structural modifications.^29^ Volcano plot analyses revealed the highest number of conformotypic peptides in the AD vs Normal pairwise comparison in CSF (Figure 2a). The number of conformotypic peptides across all pairwise comparisons in serum and CSF is summarized in Figure 2b. Proteins associated with conformotypic peptides in the LiP samples were considered candidates for structural alterations. These conformotypic peptides mapped to 14 and 42 candidate proteins exhibiting structural alterations in serum and CSF, respectively (Figure 2c). Further functional enrichment analysis using Gene Ontology (GO) and Kyoto Encyclopedia of Genes and Genomes (KEGG) pathways revealed that commonly altered structural proteins were strongly associated with biological processes such as complement activation via the alternative and classical pathways, as well as broader complement activation and coagulation cascades in both serum (Figure 2d) and CSF (Figure 2e).

**Figure 2:**
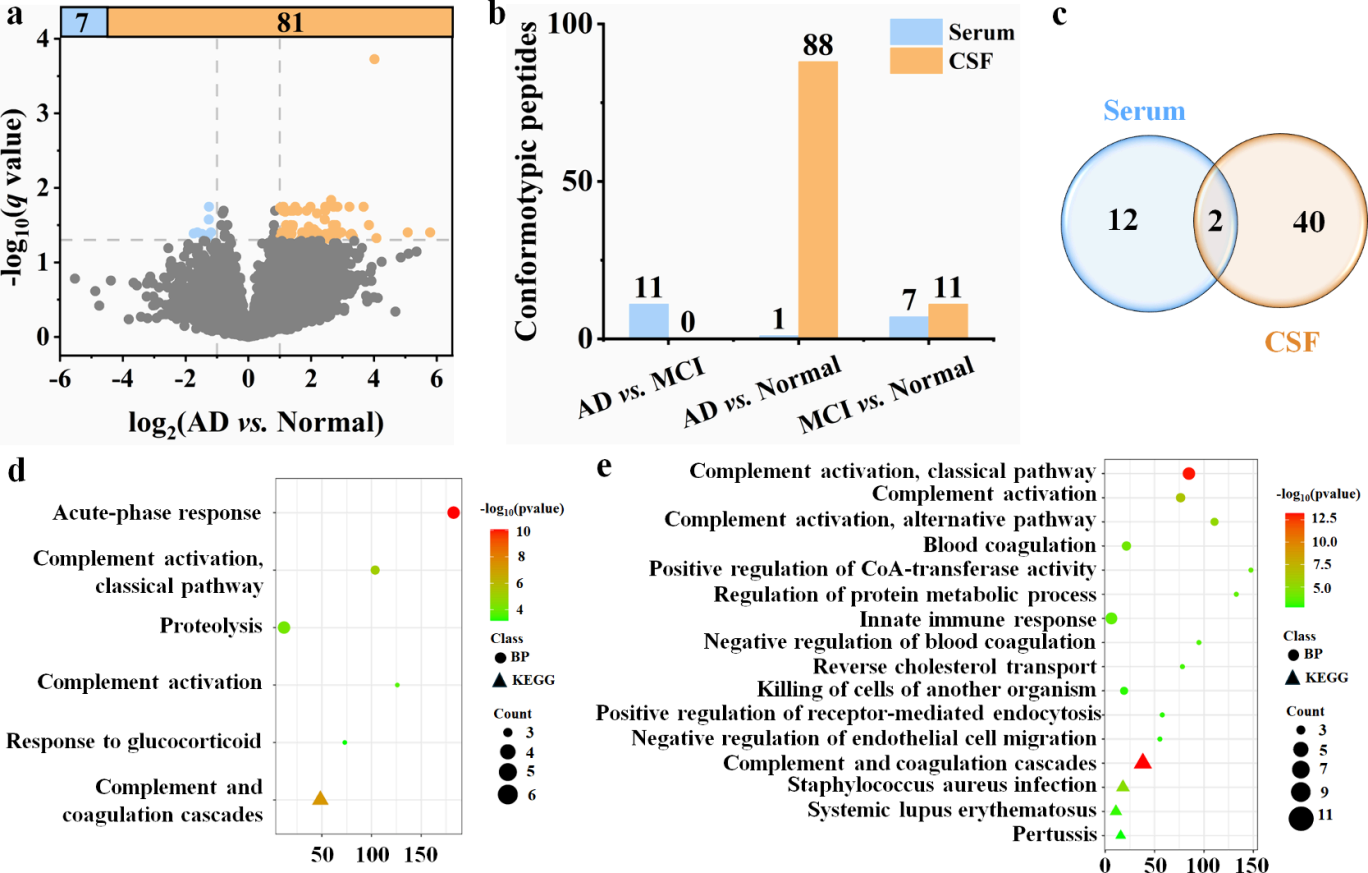
Global identification of structural changes in serum and CSF. (a) Volcano plot showing significantly altered peptides in CSF between AD and Normal groups (|log_2_(fold change)| > 1, *q* value < 0.05), with *q* values adjusted using the Benjamini–Hochberg correction to control the false discovery rate (FDR). Peptides are shown in sky blue for downregulation and light orange for upregulation. (b) Bar plot indicating the number of significantly altered peptides in pairwise comparisons across serum and CSF. (c) Venn diagram illustrates both unique and shared structural protein candidates in serum and CSF. (d, e) Bubble plots showing GO enrichment and KEGG pathway analysis for structurally altered proteins in serum (d) and CSF (e), respectively.

### Protein Structures Altered in Serum and CSF

In LiP-MS, fully tryptic (FT) peptides — generated by two trypsin cleavages — and half-tryptic (HT) peptides — typically resulting from one proteinase K and one trypsin cleavage — are expected to exhibit opposite abundance trends when structural changes occur. This inverse relationship serves as a confirmatory criterion for identifying true structural alterations.^29,30^ Comparative analysis revealed four FT/HT peptide pairs corresponding to clusterin (CLU), alpha-1-antichymotrypsin (SERPINA3), fructose-bisphosphate aldolase A (ALDOA), and cell adhesion molecule 3 (CADM3), all exhibiting significant structural changes. In serum, the CLU FT peptide (VTTVASHTSDSDVPSGVTEVVVK) was decreased in Normal relative to MCI, while its HT peptide was increased (Figure 3a). The SERPINA3 FT peptide (LYGSEAFATDFQDSAAAKK) and HT peptide (TDFQDSAAAKK) also showed reciprocal abundance patterns between AD and MCI (Figure 3b). In CSF, the ALDOA FT peptide (ALANSLACQGK) and HT peptide (SLACQGK) exhibited opposite abundance trends between MCI and Normal (Figure 3c). Similarly, the CADM3 FT peptide (SLVTVLGIPQKPIITGYK) and HT peptide (GIPQKPIITGYK) showed inverse abundance changes in AD vs Normal (Figure 3d). These results indicated the presence of structural alterations in these four proteins.

**Figure 3:**
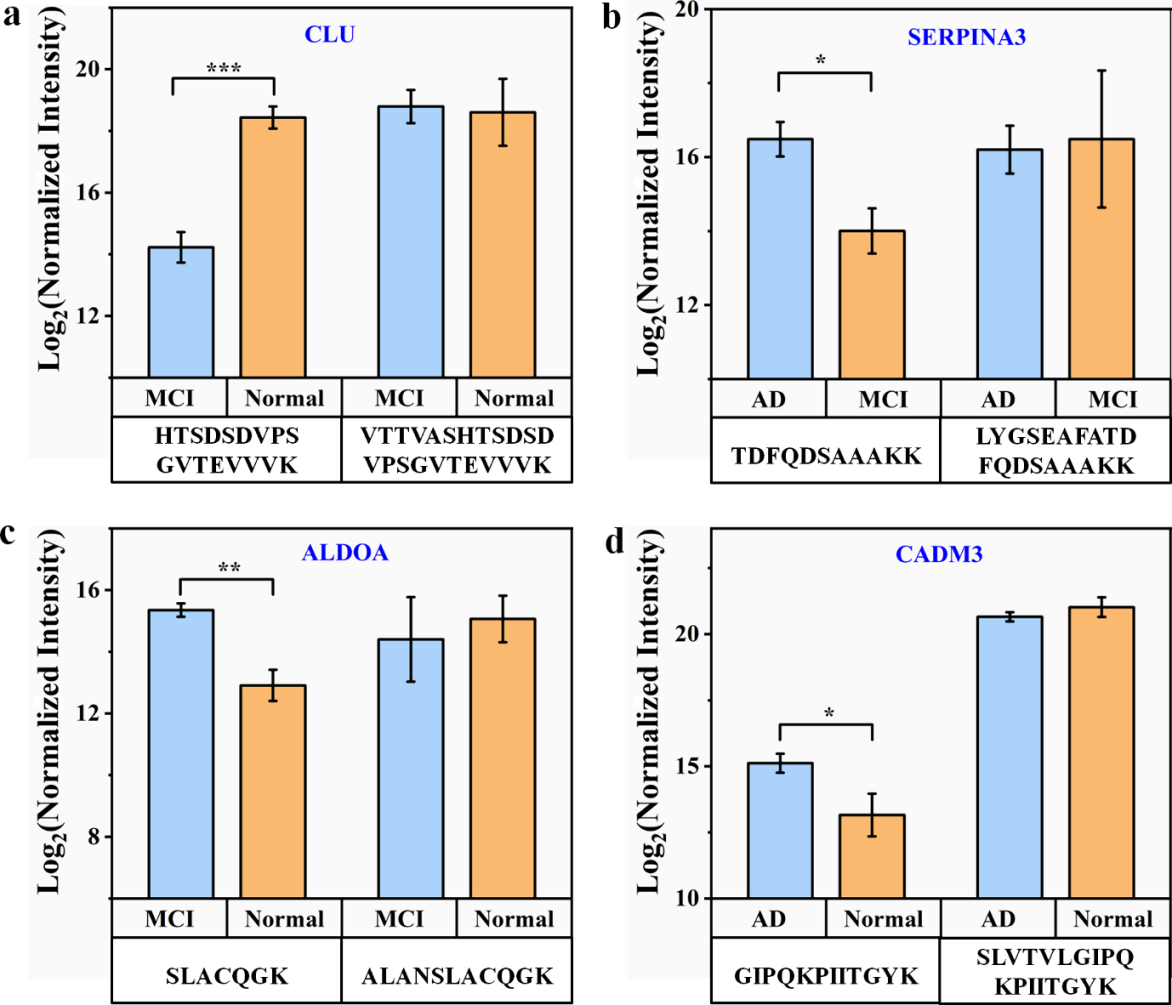
Alterations in protein structures in serum and CSF. Bar plots indicate abundance changes of CLU (a) and SERPINA3 (b) in serum, and ALDOA (c) and CADM3 (d) in CSF. Data are presented as mean ± standard deviation; *n* = 6 per group. *q* values were adjusted using the Benjamini–Hochberg correction to control FDR. Statistical significance is indicated as follows: *q < 0.05, **q < 0.01, and ***q < 0.001.

### Two Shared Structurally Altered Proteins in Serum and CSF

To identify proteins potentially associated with AD pathology, we examined those exhibiting structural changes in both serum and CSF. As illustrated in Figure 2b, two proteins — CLU and ceruloplasmin (CP) — showed structural alterations in both biofluids, and both have been previously implicated in AD pathogenesis.^31,32^ The regions of structural alteration in CLU differed between serum (Figure 4a) and CSF (Figure 4b). A similar pattern was observed for CP, with distinct structural changes identified in serum (Figure 4d) and CSF (Figure 4e). Coverage plots of conformotypic peptides revealed structural alterations in different regions of both CLU (Figure 4c) and CP (Figure 4f) across serum and CSF. Pairwise analysis showed that conformotypic peptides of CLU were upregulated in the Normal group compared with the MCI group in serum (Figure 4g) and the AD group in CSF (Figure 4h). In contrast, conformotypic peptides of CP were downregulated in the MCI group in serum (Figure 4i) and in the Normal group in CSF (Figure 4j) compared with the AD group.

**Figure 4:**
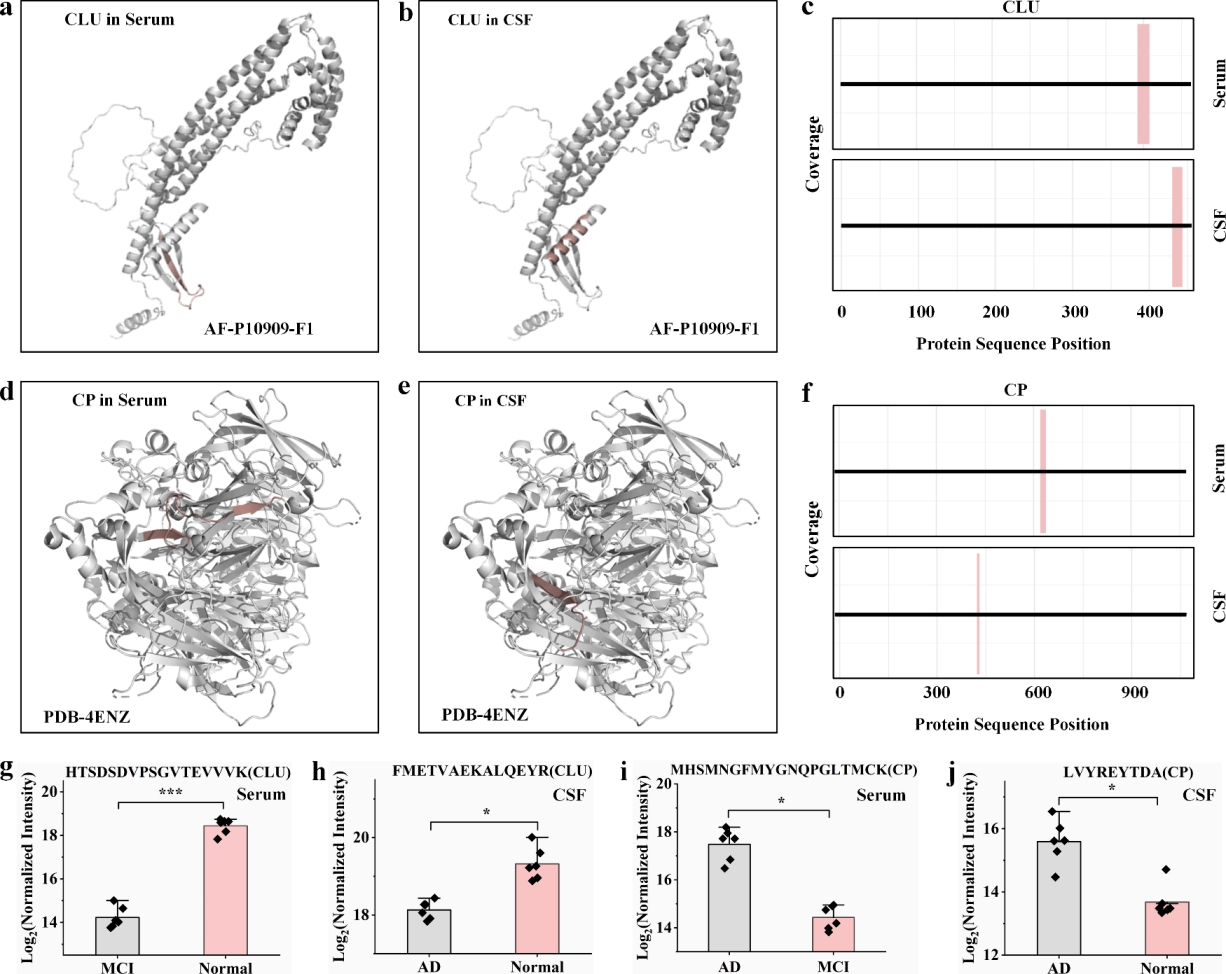
Structural changes in CLU and CP in serum and CSF. (a, b) Structures of CLU (AF-P10909) in serum (a) and CSF (b). (c) Coverage plots of conformotypic peptides for CLU in serum and CSF. (d, e) Structures of CP (PDB-4ENZ) in serum (d) and CSF (e). (f) Coverage plots of conformotypic peptides for CP in serum and CSF. (g, h) Bar plots showing abundance changes of CLU conformotypic peptides (HTSDSDVPSGVTEVVVK) in serum (g) and (FMETVAEKALQEYR) in CSF (h). (i, j) Bar plots showing abundance changes of CP conformotypic peptides (MHSMNGFMYGNQPGLTMCK) in serum (i) and (LVYREYTDA) in CSF (j). Data are presented as mean ± standard deviation; *n* = 6 per group. Statistical significance was determined using the Benjamini–Hochberg correction: *q < 0.05 and ***q < 0.001. Regions highlighted in dark salmon in the 3D protein structures indicate conformotypic peptides.

### Protein Profiles and Their Alterations during AD Progression

Accurately mapping dynamic protein changes in the paired serum and CSF samples is essential for understanding AD pathology.^33^ To trace the trajectory of protein alterations, we analyzed proteins identified at different stages of AD using Trypsin/LysC-only samples. Proteins from serum and CSF samples were independently clustered into eight groups. In serum, a greater number of proteins in clusters 4, 5, and 6 exhibited similar patterns across disease progression (Figure S9). These clusters were enriched in GO terms and KEGG pathways strongly related to the extracellular space, extracellular exosomes, complement and coagulation cascades, blood microparticles, and the collagen-containing extracellular matrix. In CSF, a greater number of proteins in clusters 6, 7, and 8 showed similar trends with disease progression (Figure 5a). Notably, cluster 8 was significantly enriched in biological processes closely linked to AD pathogenesis, including synapse organization, nervous system development, positive regulation of synapse assembly, presynaptic membrane, central nervous system development, and amyloid-beta binding.

**Figure 5:**
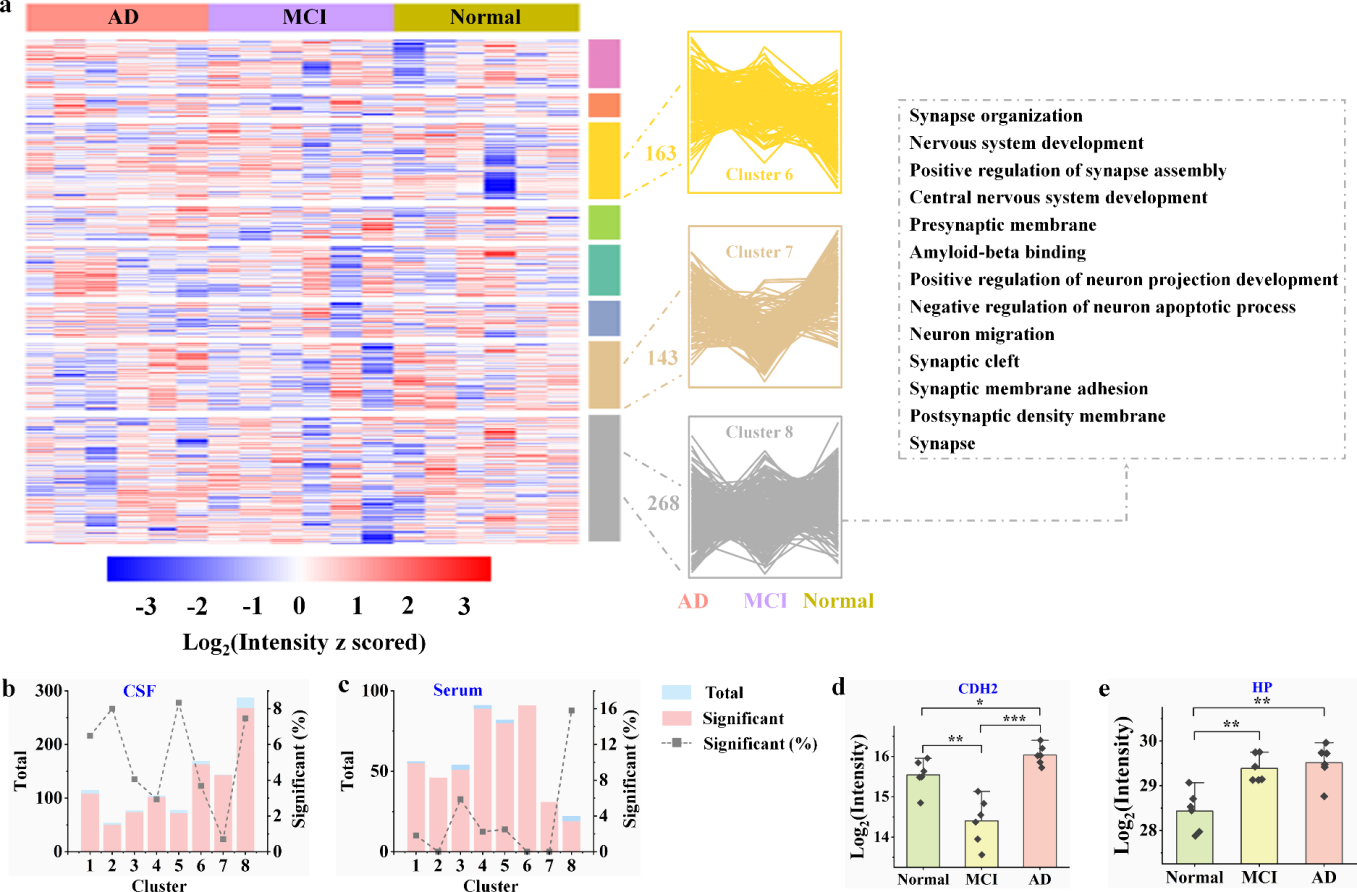
Protein profiles in serum and CSF and their alterations across different AD stages. (a) Clusters are depicted based on varying intensity profiles across AD, MCI and Normal groups. The number of proteins and selected enriched biological processes and pathways (each containing at least 5 proteins, FDR < 0.05) are indicated for each cluster. (b, c) Bar plots showing the distribution of total proteins, significantly altered proteins, and their percentages across clusters in CSF (b) and serum (c). (d, e) Bar plots showing the expression levels of CDH2 (d) and HP (e) in serum, with *q* value < 0.05 based on ANOVA analysis. *n* = 6 per group, *p* values were calculated using a two-tailed *t*-test. Statistical significance is indicated as follows: *p < 0.05, **p < 0.01, and ***p < 0.001.

To quantitatively evaluate the relationship between protein clusters and disease progression, we performed linear regression analyses on individual proteins within each cluster. The resulting p-values represent the significance of linear expression trends across disease stages. Applying a threshold of *p* < 0.05, we quantified the number and proportion of proteins in each cluster that exhibited significant linear trends in serum and CSF. As shown in Figure 5b, Cluster 8 in the CSF data set contained 268 proteins, of which 20 (7.46%) displayed significant stage-dependent expression changes, highlighting a strong association with disease progression. In contrast, only 1 out of 143 proteins (0.7%) in Cluster 7 showed a significant trend, suggesting minimal stage-related alterations in this cluster. These findings provide quantitative support that certain clusters, such as Cluster 8, are enriched for proteins linked to disease progression, whereas others, like Cluster 7, show limited stage-related expression patterns. By contrast, serum clusters overall exhibited fewer proteins with significant trends and lower proportions compared to CSF (Figure 5c).

In addition, unsupervised principal component analysis (PCA) was performed on the full proteomics data set to assess intrinsic variance and its relationship to disease state. The PCA score plots indicated that the sample types could not be clearly separated (Figure S10), while the loading plots revealed that a subset of proteins contributed to the observed variation between sample types (Figure S11). Further analysis using ANOVA (*q*-value <0.05) across the Normal, MCI and AD groups in serum identified four significantly altered proteins: cadherin-2 (CDH2), haptoglobin (HP), vitamin K-dependent protein C (PROC), and keratin, type I cytoskeletal 17 (KRT17). CDH2, a member of the cadherin family of Ca^2+^-dependent cell adhesion molecules, plays diverse roles in central nervous system development and may be involved in AD etiology.^34^ Our results indicated elevated CDH2 levels in AD patients compared to Normal controls in serum (Figure 5d), consistent with previous findings.^35^ HP expression increased progressively from normal to MCI to AD (Figure 5e), aligning with prior enzyme-linked immunosorbent assay (ELISA)-based studies in Chinese AD patients.^36^ Known for its antioxidant and anti-inflammatory properties, elevated HP levels in MCI and AD suggest a role for oxidative stress and neuroinflammation in AD pathogenesis.^37,38^ Our results also indicated elevated levels of PROC (Figure S12a) and KRT17 (Figure S12b) in the Normal group compared with the AD and MCI groups. However, evidence directly linking these proteins to AD remains limited in the current literature.

### Pairwise Analysis of Dysregulated N-Glycopeptides in Serum and CSF

Pairwise comparisons of dysregulated N-glycopeptides between groups were visualized using volcano plots (Figures 6a, 6b, and S13). The comparison between AD and Normal yielded a greater number of significantly altered N-glycopeptides in both serum and CSF. From these significantly changed N-glycopeptides, we mapped the corresponding proteins and identified eight N-glycoproteins that were shared between serum and CSF in the AD vs Normal comparison. GO enrichment analysis revealed that these N-glycoproteins are primarily associated with molecular function and biological process such as nerve growth factor binding and complement activation via the classical pathway (Figures 6c and S14).

**Figure 6:**
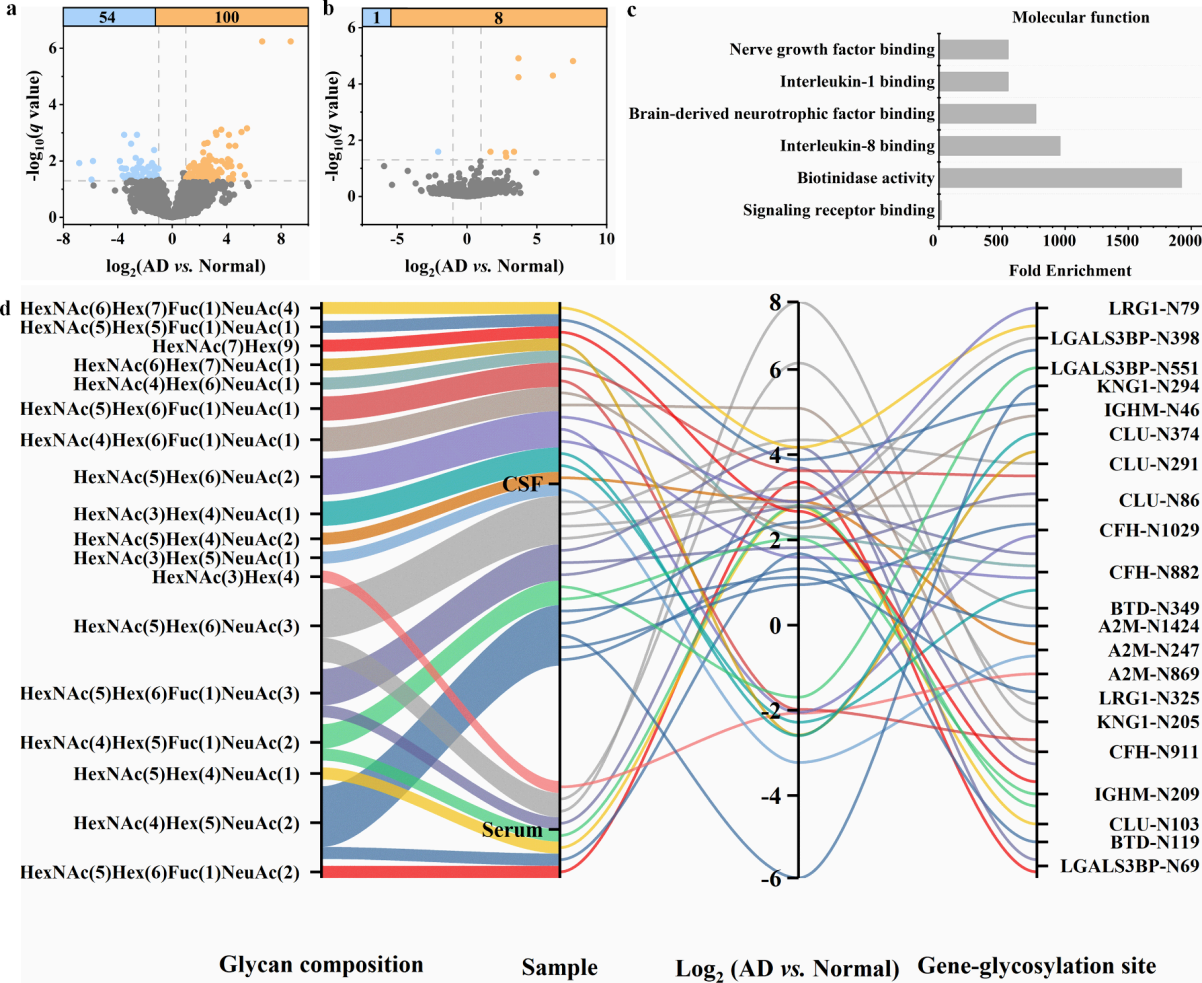
Global identification and site-specific quantitative analysis of dysregulated N-glycoproteins in serum and CSF. Volcano plots reveal dysregulated N-glycopeptides in serum (a) and CSF (b) based on pairwise comparisons between AD and Normal groups (|log_2_(fold change)| > 1, *q* value < 0.05, with *q* values adjusted using the Benjamini–Hochberg correction). N-Glycopeptides are shown in sky blue for downregulation and light orange for upregulation. GO molecular function analysis of eight dysregulated glycoproteins shared between serum and CSF (c). Parallel sets illustrate the complex relationships among glycan composition, sample type, glycopeptide expression trends, and glycosite microheterogeneity (different colored lines indicate different glycan types) (d).

We further performed site-specific quantitative analyses of the eight shared N-glycoproteins in serum and CSF, taking glycan microheterogeneity into account. Notably, N-glycopeptides with identical glycan compositions exhibited consistent upregulation trends in both serum and CSF. For example, the N-glycopeptide carrying HexNAc(4)­Hex(5)­Fuc(1)­NeuAc(2) at glycosite N209 of immunoglobulin heavy constant mu (IGHM) showed concordant upregulation in both biofluids. In contrast, N-glycopeptides with distinct glycan compositions exhibited inverse directional changes even within the same sample. This was exemplified in CSF at glycosites N882, N911, N1029 of complement factor H (CFH), and N247 of alpha-2-macroglobulin (A2M), where the corresponding N-glycopeptides displayed opposing trends. As a representative example of site-specific glycosylation changes, three distinct glycan compositions were identified at glycosite N291 of CLU in CSF. N-Glycopeptides carrying HexNAc(5)­Hex(6)­Fuc(1)­NeuAc(1) and HexNAc(5)­Hex(6)­NeuAc(3) were upregulated, whereas the N-glycopeptide with HexNAc(6)­Hex(7)­NeuAc(1) was downregulated. Interestingly, we also observed that some N-glycopeptides exhibited consistent directional changes even when both the glycan composition and the biological matrix (serum vs CSF) differed. For example, glycosite N69 of galectin-3- binding protein (LGALS3BP) showed a consistent expression trend, with glycan compositions HexNAc(5)­Hex(6)­Fuc(1)­NeuAc(2) in serum and HexNAc(5)­Hex(6)­Fuc(1)­NeuAc(3) in CSF. Additionally, glycosite N119 of biotinidase (BTD) was detected in serum, while glycosite N349 was identified in CSF, indicating that glycosite occupancy can be sample-specific.

### Correlation between Conformotypic Peptides and N-Glycosylation in Structurally Aberrant Proteins

Investigating the impact of N-glycosylation on protein structure and function offers valuable insights for developing symptomatic therapies for AD.^19^ To explore the potential relationship between protein structural alterations and N-glycosylation, we analyzed LiP cleavage sites in significantly altered HT peptides and assessed their proximity to annotated N-glycosylation sites. Two proteins, haptoglobin and kininogen-1, exhibited LiP cleavage sites within ± 60 amino acids of annotated N-glycosylation site, as reported in the UniProt database. A correlation between protein structural changes and N-glycosylation was observed. For serum haptoglobin (Figures 7a–d), a single N-glycosylation site (N184) harbored four distinct glycan compositions, and all N-glycopeptides carrying these glycans exhibited a significant positive correlation with the same LiP HT peptide, regardless of glycan composition. Additionally, a positive correlation was observed between N-glycopeptides at the N-glycosylation site N205 and the LiP HT peptide in CSF kininogen-1 (Figure S15).

**Figure 7:**
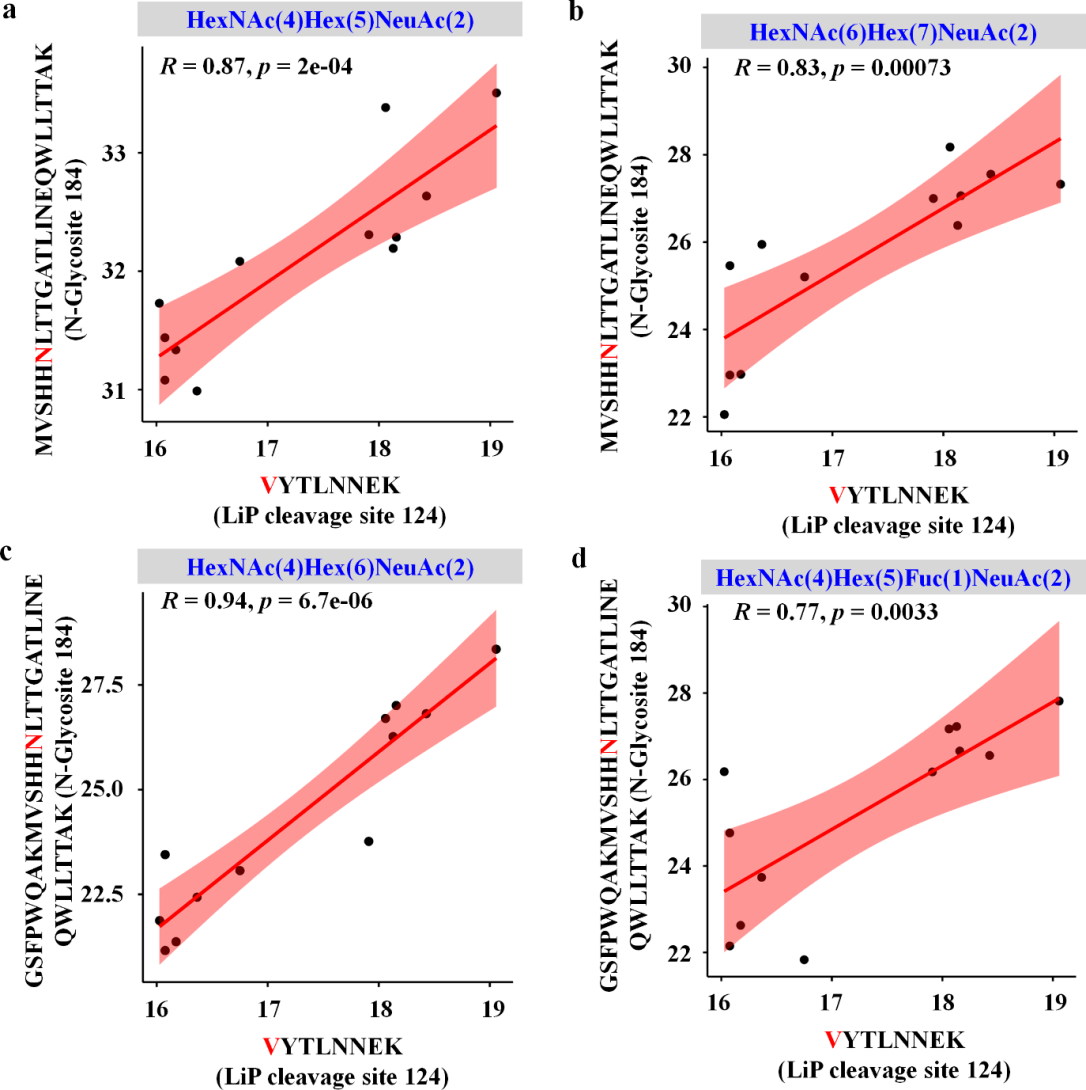
Correlation analysis between protein structural alterations and N-glycosylation in serum haptoglobin. Pearson spearman correlation analyses were performed between N-glycopeptides and LiP HT peptides in serum, focusing on four distinct glycan compositions: HexNAc(4)­Hex(5)­NeuAc(2) (a), HexNAc(6)­Hex(7)­NeuAc(2) (b), HexNAc(4)­Hex(6)­NeuAc(2) (c), and HexNAc(4)­Hex(5)­Fuc(1)­NeuAc(2) (d). The *X*-axis represents the log_2_-transformed intensity of LiP HT peptides (with the LiP cleavage site highlighted in red), and the *Y*-axis represents the log_2_-transformed intensity of N-glycopeptides (with the N-glycosylation site highlighted in red).

Collectively, these findings suggest a potential link between protein structural alterations and N-glycosylation. Although further validation is required to fully elucidate the mechanistic role of N-glycosylation in protein conformational dynamics, our preliminary observations provide a compelling foundation for future mechanistic and functional investigations.

## Discussion

In this study, we identified 54 structurally altered protein candidates in serum and CSF through pairwise comparisons across AD, MCI, and Normal groups. This data set significantly expands the current understanding of protein structural alterations associated with AD progression. The overlap of structural changes at the protein level in matched serum and CSF samples further supports the feasibility and clinical utility of using serum-based biomarkers to reflect central nervous system pathology. Moreover, GO and KEGG analyses revealed that both structurally altered proteins and dysregulated glycoproteins are enriched in the complement cascade (Figures 2d, 2e, and S14a), underscoring the critical role of immune and inflammatory pathways in AD pathogenesis.

The difference in the regions of structural alterations of CLU and CP in CSF and serum (Figures 4a and 4f) likely reflects the distinct physiological environments of the two fluids, each shaped by unique protein interactions and inflammatory contexts. In the context of AD, structural alterations in serum proteins may reflect systemic immune responses or peripheral inflammation, whereas changes in CSF proteins are more likely to represent central nervous system-specific pathology and neuroinflammation. Therefore, integrating paired serum and CSF proteomics provides a more comprehensive view of the molecular interplay between peripheral and central processes in AD. Overall, our findings highlight the value of structural proteomics in uncovering novel biomarkers and mechanistic insights into AD. The identification of shared protein-level structural changes across compartments and their association with key biological pathways offers promising avenues for early diagnosis, disease monitoring, and therapeutic targeting.

N-glycosylation is known to play critical roles in brain function and have been implicated in AD.^39^ While previous studies have suggested that AD-related proteins were glycosylated, the interplay between N-glycosylation and protein conformational dynamics in complex human samples remains poorly understood. Our data provides preliminary evidence of such relationships, as demonstrated by the positive correlation between LiP HT peptides and N-glycopeptides (Figures 7 and S15). Although further validation is needed to comprehensively elucidate the mechanistic connections between N-glycosylation and protein structural changes, these findings offer important insights into how N-glycosylation may influence protein misfolding and aggregation in AD. Future studies will adopt targeted approaches to confirm these correlations and explore their functional consequences.

In this study, analysis of demographic variables showed significant age differences across disease groups (one-way ANOVA, *p* < 0.05), while gender distribution did not differ significantly (Fisher’s exact test, p = 0.36). Normal subjects were younger than both MCI and AD groups, with no age difference between MCI and AD. Aging itself reshapes the CSF proteome, and many proteins correlate with chronological age across adulthood.^40^ Therefore, age may act as a confounding factor in interpreting disease-related biomarker changes, and future studies should control for age as a covariate or use age-matched cohorts to better distinguish disease-specific effects from those associated with aging. In addition, the small sample size limits statistical power and increases the risk of false positives and false negatives. Consistently, the PCA score plots (Figure S10) indicated that the sample types could not be clearly separated, which may reflect both the limited cohort size and overlapping proteomic features influenced by age or interindividual variability. Nonetheless, similar proof-of-concept studies in neurodegeneration have been conducted on limited cohorts, demonstrating that meaningful preliminary insights can still be obtained and later validated in larger independent cohorts.^41^ Accordingly, our findings should be interpreted as pilot and hypothesis-generating rather than definitive biomarkers. Future studies with expanded sample sizes, standardized workflows, and independent validation are needed to confirm the observed trends and strengthen the biological interpretation.

## Conclusions

This comprehensive study, leveraging paired serum and CSF samples, demonstrates that integrating protein structural data with abundance profiles significantly enhances the discovery of robust molecular biomarkers for AD. Importantly, our workflow enabled, for the first time, a parallel investigation into the relationship between protein structural changes and N-glycosylation in the context of protein misfolding diseases such as AD. Our findings underscore the value of integrating structural proteomics and N-glycoproteomics in paired serum and CSF samples from individuals with AD, MCI and normal cognition. Collectively, our study not only identifies specific molecular changes relevant to AD diagnosis and progression but also opens new avenues for AD-related research. These include the discovery of new aggregation-prone proteins and staging biomarkers, the development of potential therapeutic targets, and system-wide investigations into N-glycosylation-induced structural alterations.

## Materials and Methods

### Serum and CSF Information

The study has been approved by the University of Wisconsin Institutional Review Board and adhered to the principles of the Declaration of Helsinki. Paired serum and CSF samples from 18 individuals, including 6 individuals with Normal cognition, 6 with mild cognitive impairment and 6 with Alzheimer’s disease, were provided by the Wisconsin Alzheimer’s Disease Research Center (ADRC). Each subject signed informed consent form before participation. Samples were stored at – 80 °C until analysis. A summary of sample information, including age, gender, and disease conditions from the ADRC refers to Table S1.

### Limited Proteolysis of Serum and CSF

The protein concentration in serum and CSF samples was measured using the Pierce bicinchoninic acid Protein Assay Kit (Thermo Fisher Scientific). First, 200 μg of serum proteins and 100 μg of CSF proteins were divided into two portions: one for Trypsin/LysC-only digestion and the other for limited proteolysis (LiP) analysis. Both samples were adjusted to equal volumes using a native lysis buffer (20 mM HEPES, 150 mM KCl, 10 mM MgCl_2_) containing Roche Mini cOmplete Protease Inhibitor Cocktail (EDTA-free) at pH of 7.5. For the LiP samples, Proteinase K from *Tritirachium album* (Sigma-Aldrich) was added at an enzyme-to-substrate ratio of 1:100 (w/w) and incubated at room temperature for 5 min. Digestion was then halted by transferring the mixture into a new tube containing guanidine hydrochloride powder to a final concentration of 7.6 M, followed by heating the mixture for 3 min in a water bath over 95 °C. Trypsin/LysC-only samples were added with an equal volume of cold water and subjected to the same heating process. After cooling, both sample groups were subjected to complete tryptic digestion. The proteins were reduced with dithiothreitol to a final concentration of 5 mM for 30 min at 37 °C, followed by alkylation with iodoacetamide at a final concentration of 15 mM for 45 min at room temperature in the dark. To prepare for digestion, the samples were diluted with 0.1 M ammonium bicarbonate to achieve a final guanidine hydrochloride concentration of 0.5 M. Trypsin/LysC (Promega) was then added at an enzyme-to-substrate ratio of 1:50 (w/w), and digestion proceeded overnight at 37 °C. Following digestion, the samples were acidified with trifluoroacetic acid to lower the pH below 3 and desalted using Sep-Pak C18 cartridges (Waters). The concentration of peptides was determined using the Pierce Quantitative Colorimetric Peptide Assay (Thermo Fisher Scientific).

### Database Searching

All data were searched using MSFragger (version 4.0),^42,43^ Philosopher (version 5.1.0), and FragPipe (version 21.1). The reviewed *H. sapiens* proteins and common contaminant sequences were downloaded from UniProt (downloaded on December 11, 2023, UP000005640, 40,924 entries, including 20,462 decoys) were used. Precursor and fragment mass tolerance was set at ± 20 PPM and 20 PPM, respectively. Protease K and trypsin were set as enzymes with semicleavage rules in the LiP samples, while strict trypsin rule was used for the Trypsin/LysC samples, two missed cleavages were allowed for both LiP and Trypsin/LysC-only samples. Variable modifications of methionine oxidation and N-terminal plus fixed modification of cysteine carbamidomethylation were assigned. Unless explicitly noted otherwise, all these parameters are the same for the following three aspects and used for the default.

### Data Analysis and Visualization

#### Peptide Filtering and Normalization

Peptides filtering and normalization steps were applied separately to the Trypsin/LysC-only treated data and the Proteinase K treated LiP data prior to further analysis. Albumin and Biognosys peptides from the Trypsin/LysC-only group, as well as peptides belonging to albumin and Biognosys in the LiP data, were removed. Peptides and proteins detected in fewer than 50% of samples within each cohort group were excluded from further analysis. This step was performed using Perseus (version 1.6.15.0).^44^ The mean intensity of the two technical replicates was calculated for both LiP and Trypsin/LysC-only data sets and used as the representative value for each sample. LiP peptide data were normalized based on significant changes in protein abundance, defined by *p* value <0.05 and |log_2_(fold change)| > 1. *p* values were calculated using a two-sided *t*-test assuming equal variance. For proteins that did not exhibit significant changes in abundance, a normalization factor of 1 was applied.^45^ Normalized intensities were then log_2_-transformed in Perseus. Missing values were imputed using the “Replace missing values from normal distribution” feature. Two-sample tests were performed, and the resulting data were used for conformotypic peptide matching, with thresholds of |log_2_(fold change)| > 1, and *q* value <0.05 (*q* values were adjusted using the Benjamini–Hochberg correction to control the FDR). Proteins associated with conformotypic peptides were deemed structurally altered.

#### Demographic and Glycoproteomics Data Analysis

Demographic variables, including age and gender, were analyzed using GraphPad Prism (version 10). Age differences among disease groups were assessed using one-way ANOVA for continuous variables, while gender distribution was evaluated using Fisher’s exact test for categorical variables. For N-glycoproteomics data analysis, the data set was refined by filtering out glycans with a q-value greater than 0.01 and entries with a Hyperscore less than 10. Label-free quantification intensities were log_2_-transformed in Perseus (version 1.6.15.0), and missing values were imputed using the “Replace missing values from normal distribution” feature prior to further analysis.

#### Clustering and Visualization

For clustering analysis, protein expression data were log_2_-transformed, and missing values were imputed using the “Replace missing values from normal distribution” function in Perseus. The resulting data set was standardized using Z-score transformation prior to further analysis. Hierarchical clustering and heatmap visualization were performed using the pheatmap package in R, applying default parameters (Euclidean distance and complete linkage). Stage-dependent trends in cluster expression were further evaluated using linear regression analysis.

GO and KEGG pathway analyses were performed by the Database for Annotation, Visualization and Integrated Discovery (DAVID). Protein structure visualization was conducted using PyMOL (version 2.5.4). Volcano plots, bar plots, and parallel sets were generated using Origin (version 2020). Enrichment bubble plots of structurally altered proteins and pearson spearman correlation analyses were conducted via the free online platform (https://www.bioinformatics.com.cn).^46^ Peptide coverage plots, PCA score and loading plots, and cluster analyses were performed in the R (version 4.3.2).

## Supplementary Material



## Data Availability

The mass spectrometry proteomics data have been deposited to the ProteomeXchange Consortium via the PRIDE partner repository with the data set identifier PXD059280.

## Notes

Chemicals and materials; HILIC enrichment; HpH fractionation; LC-MS/MS analysis of pooled sample for library generation under DDA; LC-MS/MS analysis of individual serum and CSF under DIA; LC-MS/MS analysis for N-glycoproteomic profiling under DDA; DDA data search for library generation; DIA data search; N-glycoproteomic data search; top 14 high abundance protein depletion method; FAIMS-DIA-MS conditions optimization; comparison of library-based and library-free for DIA MS data acquisition; Figures S1–S15; and Table S1 (PDF)
