# Supplementary material for: Integrated Structural and Glycoproteomic Profiling Reveals Protein Conformational Remodeling and Biomarkers Across Alzheimer’s Disease Progression
Source: ACS Cent Sci. 2026 Jan 2;12(1):75–87. doi: 10.1021/acscentsci.5c02048 (PMC12856674; doi:10.1021/acscentsci.5c02048)
Supplement: Supplementary file 1 [file oc5c02048_si_001.pdf]

Supporting Information

Integrated Structural and Glycoproteomic Profiling Reveals Protein Conformational Remodeling and Biomarkers Across Alzheimer's Disease Progression

Haiyan Lu[1], Ching-Yuan Yang[2], Hua Zhang[1], Xudong Shi[3], Penghsuan Huang[4], Peng-Kai Liu[2], Zicong Wang[1], Sanjay Asthana[5], Cynthia Carlsson[5], Ozioma Okonkwo[5], and Lingjun Li[1,2,4,6,7*]

[1]School of Pharmacy, University of Wisconsin-Madison, Madison, WI 53705, USA

[2]Biophysics Graduate Program, University of Wisconsin-Madison, Madison, WI 53706, USA

[3]School of Medicine and Public Health, University of Wisconsin-Madison, Madison, WI 53792, USA

[4]Department of Chemistry, University of Wisconsin-Madison, Madison, WI 53706, USA

[5]School of Medicine and Public Health, University of Wisconsin, Madison, WI 53705, USA

[6]Lachman Institute for Pharmaceutical Development, School of Pharmacy, University of Wisconsin-Madison, Madison, WI, 53705, USA

[7]Wisconsin Center for NanoBioSystems, School of Pharmacy, University of Wisconsin-Madison, Madison, WI 53705, USA.

***Corresponding author email:** lingjun.li@wisc.edu

This PDF file includes:

Chemicals and materials

HILIC enrichment

HpH fractionation

LC-MS/MS analysis of pooled sample for library generation under DDA

LC-MS/MS analysis of individual serum and CSF under DIA

LC-MS/MS analysis for N-glycoproteomic profiling under DDA

DDA data search for library generation

DIA data search

N-glycoproteomic data search

Top 14 high abundance protein depletion method

FAIMS-DIA-MS conditions optimization

Comparison of library-based and library-free for DIA MS data acquisition

Figures S1 to S15

Table S1

Chemicals and materials

Optima UPLC-grade acetonitrile (ACN), Optima UPLC grade water, Optima LC/MS grade formic acid (FA), ammonium bicarbonate (NH_4HCO_3), potassium chloride (KCl), and magnesium chloride hexahydrate ($MgCl_2·6H_2O$) were obtained from Fisher Scientific (Hampton, NH). 3 kDa molecular weight cut-off filters were sourced from Millipore Sigma (Burlington, MA). Proteinase K from *Tritirachium album*, iodoacetamide, trifluoroacetic acid (TFA), HEPES, guanidinium hydrochloride, ammonium formate, and dithiothreitol were purchased from Sigma-Aldrich (St. Louis, MO). EDTA-free Protease Inhibitor Cocktail was acquired from Roche (Basel, Switzerland). High-Select™ Top14 Abundant Protein Depletion Midi Spin Columns, pierce BCA protein assay kit and quantitative colorimetric peptide assay were obtained from Thermo Fisher Scientific (Waltham, MA). Mass spectrometry grade Trypsin/Lys-C were purchased from Promega (Madison, WI). Sep-Pak C18 cartridges and Bridged Ethylene Hybrid (BEH) C18 particles were purchased from Waters Corporation (Milford, MA). PolyHYDROXYETHYL A material was purchased from PolyLC Inc. (Columbia, MD).

HILIC enrichment

N-glycopeptide enrichment was performed using hydrophilic interaction liquid chromatography (HILIC) enrichment. A 200 µL pipette tip was tightly packed with 3 mg of cotton. HILIC beads (PolyHYDROXYETHYL A, 12 µm, 300 Å, PolyLC) were suspended in 1% TFA and activated by vortexing for 15 minutes. The activated bead slurry was then loaded to the cotton-packed tip, and excess solvent was removed by centrifugation at 200g for 2 minutes. The beads were conditioned three times each with 300 µL 1% TFA and binding buffer (1% TFA in 80% acetonitrile (ACN)). For enrichment, 500 µg of serum tryptic peptides and 200 µg of CSF tryptic peptides were resuspended in 300 µL of binding buffer and loaded onto the beads, with a beads-to-peptides mass ratio of 30:1. The flow-through was collected and reloaded onto the tip five additional times to ensure maximal glycopeptide binding (centrifugation at 100g for 5 minutes per loading). The tip was subsequently washed six times with 300 µL of washing buffer (1% TFA in 80% ACN) to remove non-glycopeptides. Bound

glycopeptides were eluted in four sequential fractions using 300 μL of elution solvents with decreasing concentrations of ACN: 70% ACN with 0.1% formic acid (FA), 60% ACN with 0.1% FA, 50% ACN with 0.1% FA, and 0.1% FA. Each elution step was performed at 200g for 2 minutes. The eluates were collected separately and dried in vacuo for subsequent LC-MS/MS analysis.

HpH fractionation

For library generation, both CSF and serum samples have three condition pools (AD, MCI, and control) of the LiP-treated and the Trypsin/LysC-only were prepared (in total 6 pools, 300 μg each pool). These pools were created by combining equal volumes from each biological sample. Each pool was fractionated by off-line HpH fractionation using a Waters Alliance e2695 high-performance liquid chromatography (HPLC) system equipped with a C18 reversed-phase column (2.1 × 150 mm, 5 μm, 100 Å, Phenomenex). The separation was achieved using mobile phase A, consisting of 10 mM ammonium formate at pH 10, and mobile phase B, composed of 90% ACN and 10 mM ammonium formate at pH 10. The samples were dissolved in mobile phase A and separated according to the following gradient: 1% B (0–5 min), 1–40% B (5–50 min), 40–60% B (50–54 min), 60–70% B (54–58 min), and 70–100% B (58–59 min). A total of 30 fractions were collected at 2-minute intervals. Non-adjacent fractions were then combined into 8 pooled samples and dried in vacuo. Afterwards, indexed Retention Time (iRT) peptides were added to samples for the prediction of retention time before LC-MS/MS analysis.

LC-MS/MS analysis of pooled sample for library generation under DDA

Each fraction (1 μg) added with iRT peptides was analyzed using an Orbitrap Exploris 480 mass spectrometer coupled with a Vanquish Neo UHPLC and a FAIMS Pro Duo interface, operating in DDA mode. Peptides were loaded onto a 75-μm inner diameter microcapillary column, which was custom-packed with 15 cm of Bridged Ethylene Hybrid (BEH) C18 particles (1.7 μm, 130 Å, Waters). The mobile phase flow rate was set at 300 nL/min, with buffer A of 0.1% FA in water and buffer B of 0.1% FA in 80% ACN. Peptide separation was performed using a 123-minute gradient: 3–37.5% buffer B over 102 minutes, 37.5–90% buffer

B for 30 s, 90% buffer B for 9.5 min, 90–100% buffer B for 30 s, and 100% buffer B for 10.5 min. An ionization voltage of 1900 V and an ion transfer tube temperature of 305°C were applied. Full scan spectra were acquired in the *m/z* range of 350–1500 with an Orbitrap resolution of 60,000. The automatic gain control (AGC) target was set to 300%, with a maximum injection time (IT) of 25 milliseconds (ms) and an RF lens voltage of 50%. Dynamic exclusion was 30s duration, the data-dependent mode was set to cycle time, and the time between master scans was 1.5 seconds. The precursor ions with charges between 2 and 6, exceeding a signal threshold of 5.0×10^3, were selected for fragmentation within a 2 Da isolation window. Fragmentation was achieved using higher energy collisional dissociation (HCD) with a normalized collision energy of 28%, and fragment ion spectra were acquired with an Orbitrap resolution of 15,000 at *m/z* 120 and a maximum IT of 40 ms. For FAIMS, a gas flow rate of 4.2 L/min was maintained, with combinations of -45V and -65V.

LC-MS/MS analysis of individual serum and CSF under DIA

Peptides (1 μg) added with iRT peptides were conducted using an Orbitrap Exploris 480 mass spectrometer coupled with a Vanquish Neo UHPLC and FAIMS Pro Duo interface under DIA mode. Peptides were analyzed using a length of 15 cm custom-packed BEH C18 column (1.7 μm, 130 Å, Waters) with a 90-minute gradient at a flow rate of 300 nL/min. The gradient consisted of 3–37.5% buffer B over 69 minutes, followed by 37.5–90% buffer B for 30 s, 90% buffer B for 9.5 min, 90–100% buffer B for 30 s, and 100% buffer B for 10.5 min. Full MS spectra were acquired in the *m/z* range of 380–985 with a resolution of 60,000, an normalized AGC target of 300%, and a maximum IT of 25 ms. The full MS scan was followed by 60 DIA scans and the isolation windows were 10 *m/z* wide with a 1 *m/z* overlap. Fragmentation was performed in the *m/z* range of 145–1450, with a resolution of 15,000, a normalized AGC target of 2000%, a normalized collision energy of 28%, and a maximum IT of 40 ms. FAIMS settings included compensation voltages of −45 V and −65 V, with a gas flow rate of 4.2 L/min.

LC-MS/MS analysis for N-glycoproteomic profiling under DDA

Enriched N-glycopeptides were analyzed by LC-MS/MS analysis on Orbitrap Fusion Lumos

Tribrid Mass Spectrometer (Thermo Fisher Scientific) coupled with a Dionex UltiMate 3000 UPLC system. Samples were dissolved in 0.1% FA in water and loaded onto a 75 μm inner diameter homemade microcapillary column, packed with 15 cm of BEH C18 particles (1.7 μm, 130 Å, Waters). The mobile phase A was 0.1% FA in water and mobile phase B was 0.1% FA in 80% ACN. A 136-minute gradient was employed for the separation of the *N*-glycopeptides at a flow rate of 300 nL/min. The mobile phase B was held at 3% for the first 18.3 min, then ramped to 37.5% over 82 min. The mobile phase B was further increased from 40% to 60% in 100.5–110 min, then 60% to 99% in 30 s, and then kept constant at 99% for 10 min. Subsequently, the mobile phase decreased back to 3% phase B and held constant for 16 min. Full MS scans of *N*-glycopeptides precursors were conducted within the mass range of *m/z* 400 to 2000 with a resolution of 120,000, an RF lens 30% and a maximum IT of 100 ms. For DDA of MS/MS spectra, the resolution was set to 60,000 at *m/z* 110. MS/MS data were collected with a fixed cycle time of 3 s. The most intense peaks above a signal threshold of 2.5×10^4 were selected within an isolation window of 1.6 Da, a normalized AGC target of 60%, and a maximum IT of 200 ms for fragmentation. Step HCD was used with collision energies of 27%, 30%, and 33%.

DDA data search for library generation

The DDA raw files from pooled samples subjected to HpH fractionation were searched centering on the default workflow. Generate library from search results was enabled and Biognosys iRT kit was chosen to perform retention time calibration.

DIA data search

The DIA raw files were searched centering on the DIA_SpecLib_Quant workflow with default search parameters. The MSFragger-DIA analysis was performed using a spectral library built from fractionated DDA data. For DIA data, DIA-NN (version 1.8.2) within the FragPipe (version 21.1) was used. Biognosys iRT standards were used for retention time calibration.

N-glycoproteomic data search

The N-glycoproteomic data was searched with the glyco-N-LFQ workflow. A glycan database of human 252 unique N-glycan masses was used, with glycan composition assignments filtered to 1% FDR and a 50 ppm mass tolerance. For MS1 quantification, the minimum required number of ions was set to 2, match-between-runs (MBR) and normalized intensity across runs were enabled, MBR using ion FDR was set at 1%.

Top 14 high abundance protein depletion method

To improve the detection of lower-abundance proteins, serum immunodepletion was performed using Thermo Scientific's High-Select Top14 Abundant Protein Depletion Midi Spin Columns, following the manufacturer's protocol. Briefly, 100 μL of serum was incubated in the column for 10 minutes with gentle end-over-end mixing at room temperature. After incubation, the depleted flow-through (FT) was collected by centrifugation at 1,000 × g for 2 minutes, then concentrated using 3 kDa molecular weight cut-off filters (MilliporeSigma) via centrifugation at 12,000 × g for 30 minutes. A buffer exchange was performed using native lysis buffer (20 mM HEPES buffer, 150 mM KCl, 10 mM $MgCl_2$), containing Roche Mini cOmplete Protease Inhibitor Cocktail (EDTA-free) at pH of 7.5.

FAIMS-DIA-MS conditions optimization

To define the optimal compensation voltage (CV) range for serum proteomic analyses, we conducted an analysis of tryptic peptides derived from pooled serum samples, encompassing subjects from Normal, MCI, and AD groups. The number of unique protein groups and peptides under different CV settings were evaluated. The results showed that analyses with FAIMS resulted in more protein identifications compared to analyses without FAIMS (**Figure S6a**). While analyses without FAIMS identified more peptides than any single CV setting, the combination of -45V and -65V showed comparable peptides identifications to analyses without FAIMS (**Figure S6b**). Examination of the precursor ion charge states distributions revealed a higher prevalence of multiply charged features within the selected CV range of -40V to -65V (**Figure S6c**). This indicates that less desirable single charged ions (+1) features can be largely excluded from proteomic analyses when using FAIMS device. Additionally, we found that +2

and +3 ions exhibited opposite trends between -40 V and -65 V, suggesting different transmission mechanisms for +2 and +3 ions. The Venn diagrams indicate high reproducibility in FAIMS-DIA acquisition (**Figure S6d**). Overall, our results highlight that the utility of FAIMS-DIA can significantly enhance both the depth and reproducibility of AD serum proteomics.

Comparison of library-based and library-free for DIA MS data acquisition

Spectral libraries can be created from DDA data, with offline fractionation DDA has historically been considered the "gold standard" for libraries creation. To maximize proteome coverage, we compared library-based and library-free analysis methods using spectra generated from offline HpH fractionation samples acquired via DDA and sequence libraries, respectively. Overall, our results indicated that DIA library-based analysis resulted in more protein identifications (except for analyses without FAIMS and at -40 V) and peptide identifications compared to the library-free method (**Figure S7**). Based on the unique protein and peptide identifications, we selected the combination of -45 V and -65 V for the global characterization of proteomics in paired CSF and serum samples.

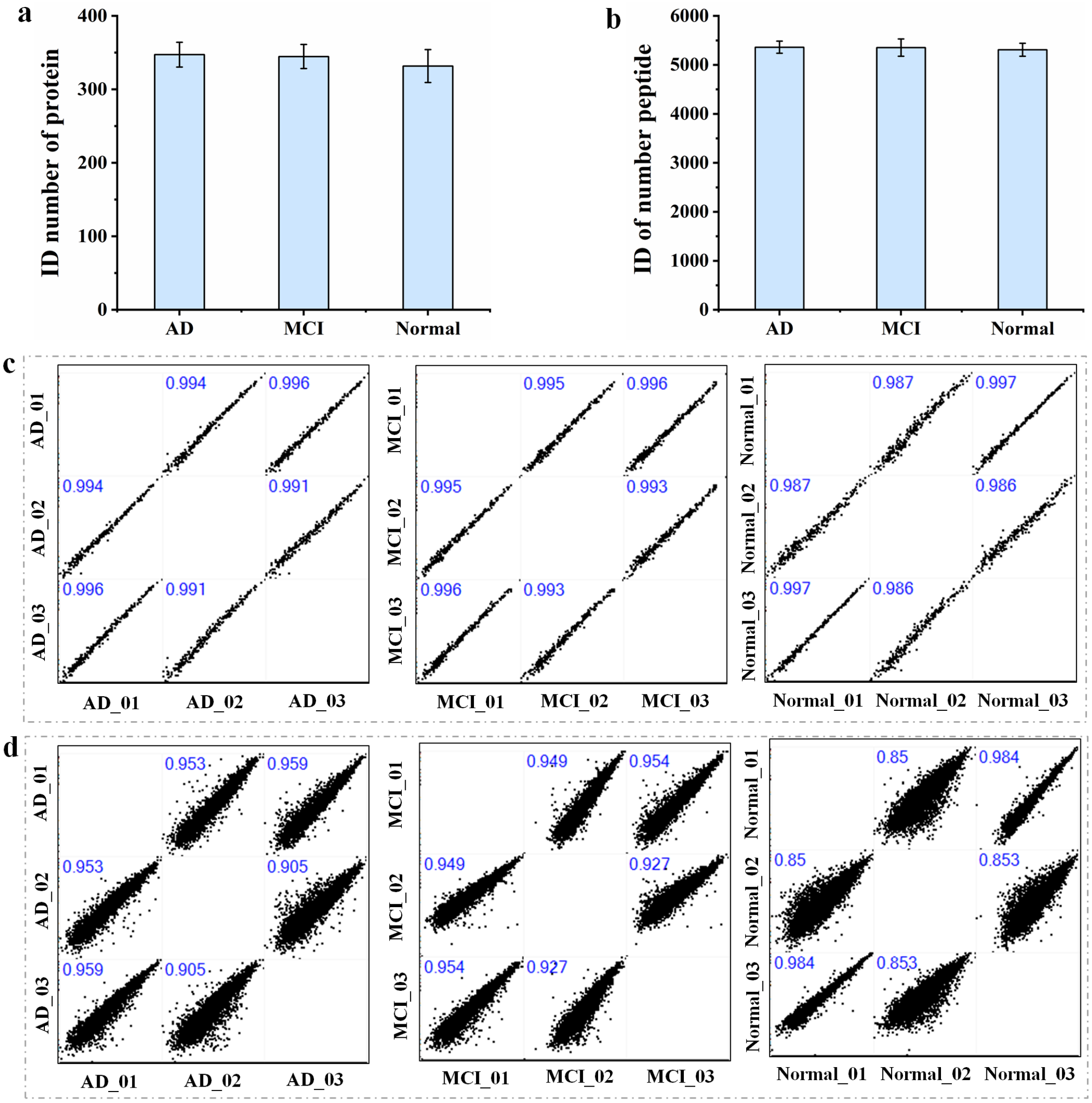

Figure S1. Evaluation of the reproducibility of Top14 Abundant Protein Depletion Columns. Identification of proteins (a) and peptides (b) within AD, MCI and Normal serum samples. Pearson correlation coefficient was calculated at both the protein level (c) and the peptide level (d). Three technical replicates were performed for each group.

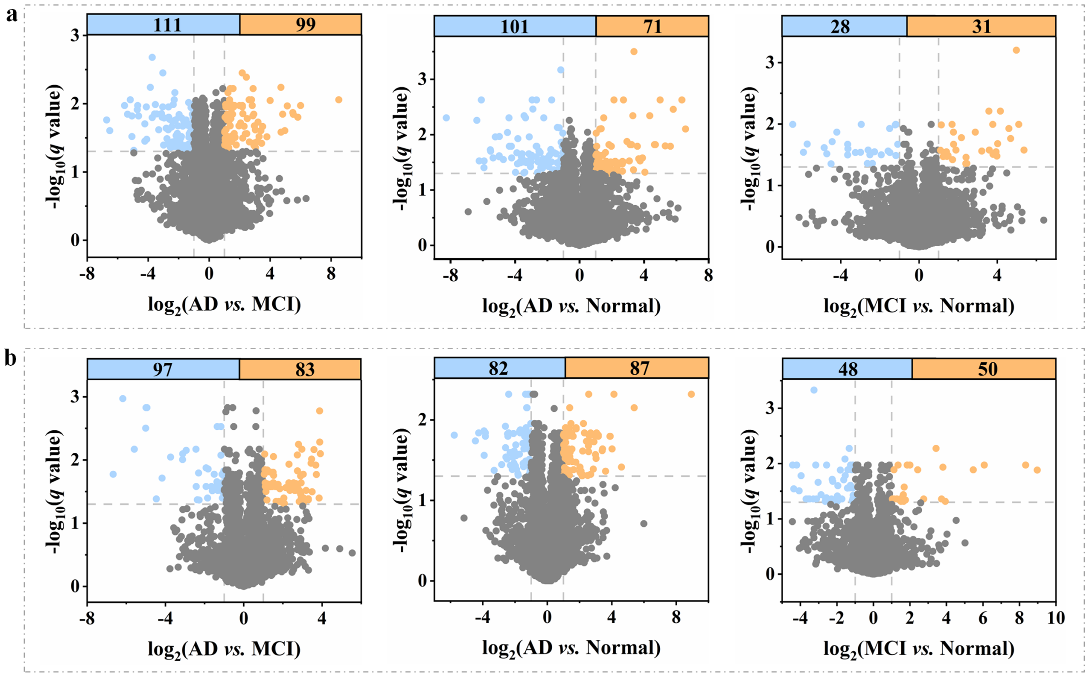

Figure S2. Volcano plots indicate the identified conformotypic peptides (with a |log2(fold change)| >1 and q values <0.05) through pairwise analysis across AD *vs.* MCI, AD *vs.* Normal, and MCI *vs.* Normal, comparing depleted (a) and undepleted (b) groups, with q values adjusted using the Benjamini-Hochberg correction, peptides are shown in blue for downregulation and orange for upregulation.

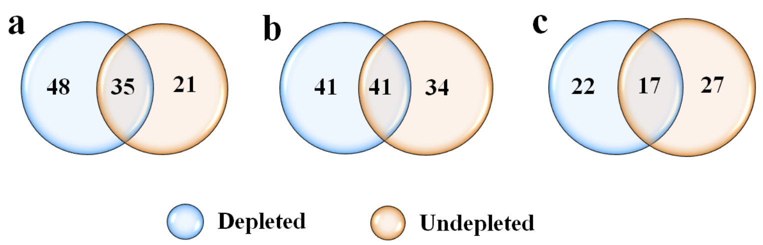

Figure S3. Venn diagrams show the common and unique structural variants between depleted (sky blue) and undepleted (light orange) groups: (a) AD *vs.* MCI, (b) AD *vs.* Normal, and (c) MCI *vs.* Normal in LiP-MS group.

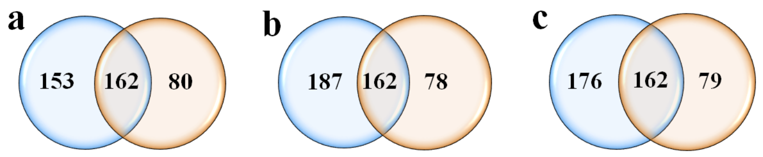

Figure S4. Venn diagrams show the common and unique proteins identified between depleted (sky blue) and without undepleted (light orange) groups: (a) AD, (b) MCI, and (c) Normal in Trypsin/LysC-only group.

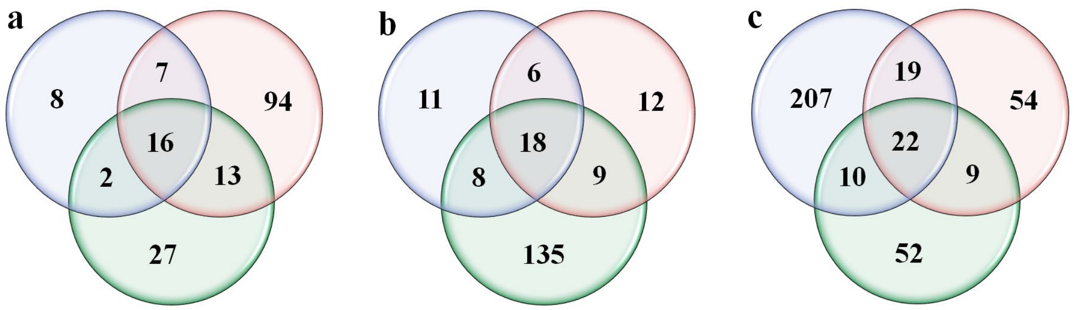

Figure S5. Venn diagrams show the overlap of proteins identified in flow-through samples across three technical replicates for (a) AD, (b) MCI, and (c) Normal groups.

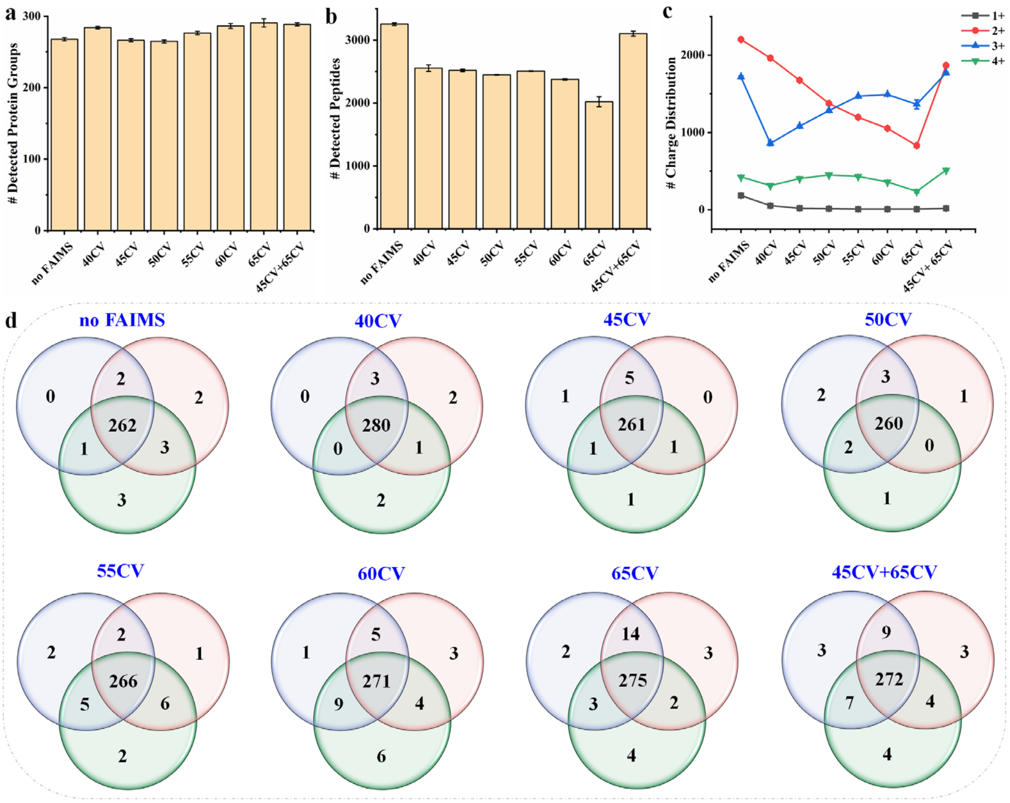

Figure S6. Characterization of FAIMS-DIA settings. The number of detected unique protein groups (a) and peptides (b) under different CVs conditions. (c) Distributions of precursor charge states under different CVs conditions. (d) Venn diagrams illustrate the technical reproducibility across three technical replicates under different CVs conditions.

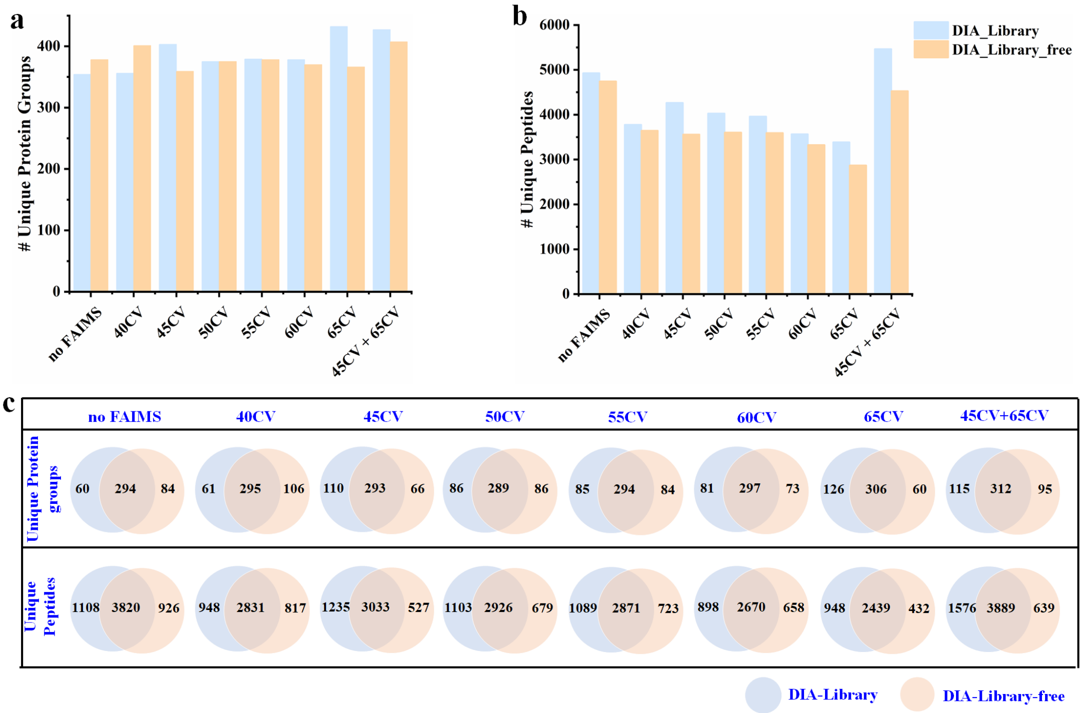

Figure S7. Comparison analysis of DIA-Library and DIA-Library-free results searched by DIA-NN under different CVs conditions. Identification of unique protein groups (a) and peptides (b). (c) Venn diagrams display the number of unique protein groups and peptides exclusively detected in either DIA-Library or DIA-Library-free approach.

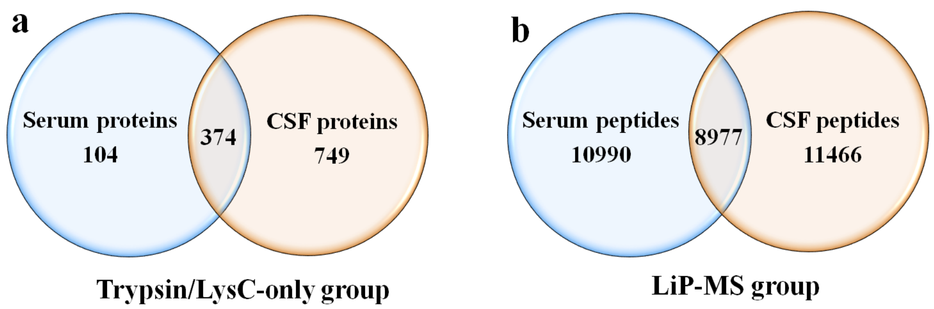

Figure S8. Identification numbers of proteins (a) and peptides (b) in serum and CSF samples.

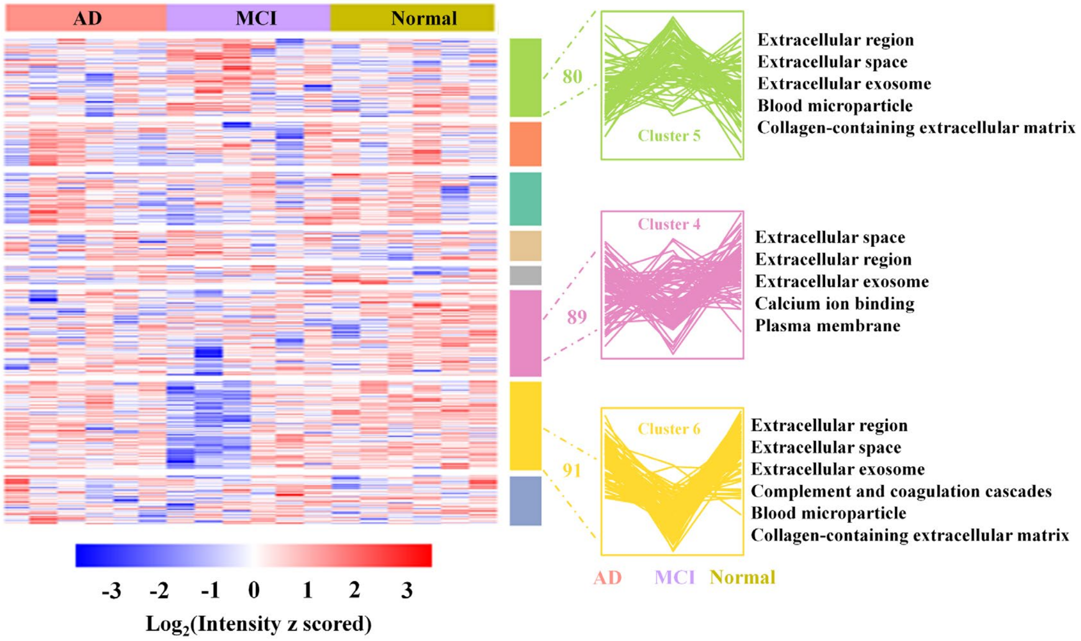

Figure S9. Protein profiles in serum and their alterations across different AD stages. (a) Hierarchical clustering of 462 proteins identified, with proteins in clusters 4, 5 and 6 showing a greater number of proteins with similarities. The number of proteins and selected enriched biological processes and pathways are indicated for each cluster (each term containing at least 20 proteins, FDR < 5.0×10^{-6}).

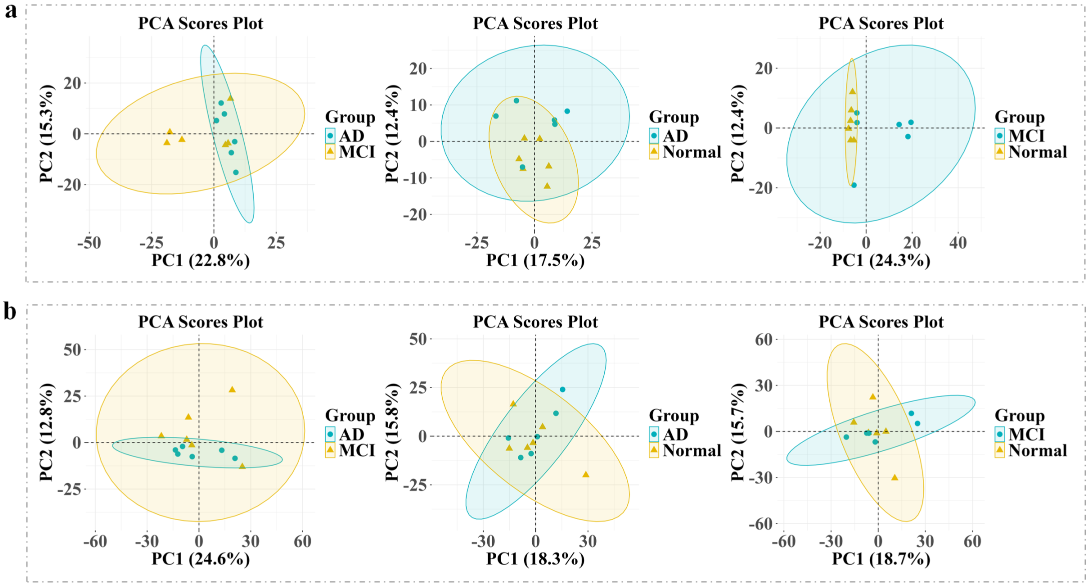

Figure S10. The PCA score plots of full proteomics dataset across pairwise analysis in serum (a) and CSF (b).

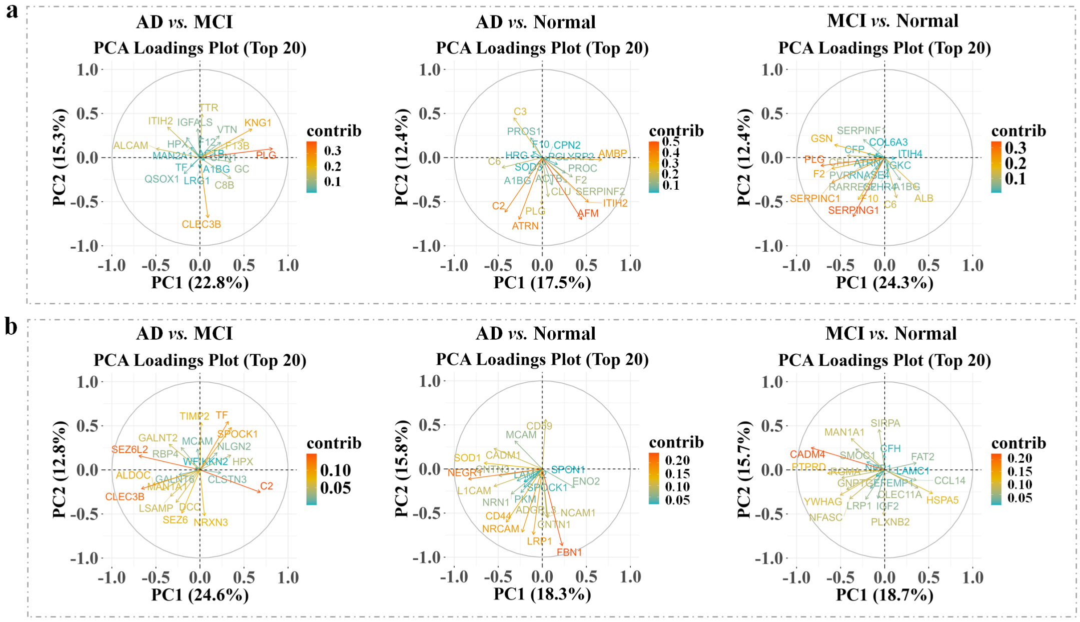

Figure S11. PCA loading plots of the full proteomics dataset from pairwise analyses in serum (a) and CSF (b). Only the top 20 contributing proteins are displayed for readability.

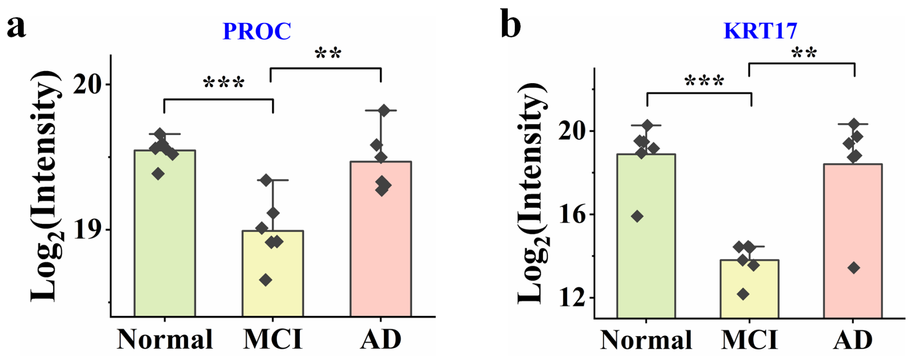

Figure S12. Bar plots show the expression levels of PROC (a) and KRT17 (b) with q-value < 0.05 via ANOVA analysis in serum, n = 6 per group, p-values calculated by two-tailed *t*-test, $^{**}p < 0.01$, and $^{***}p < 0.001$.

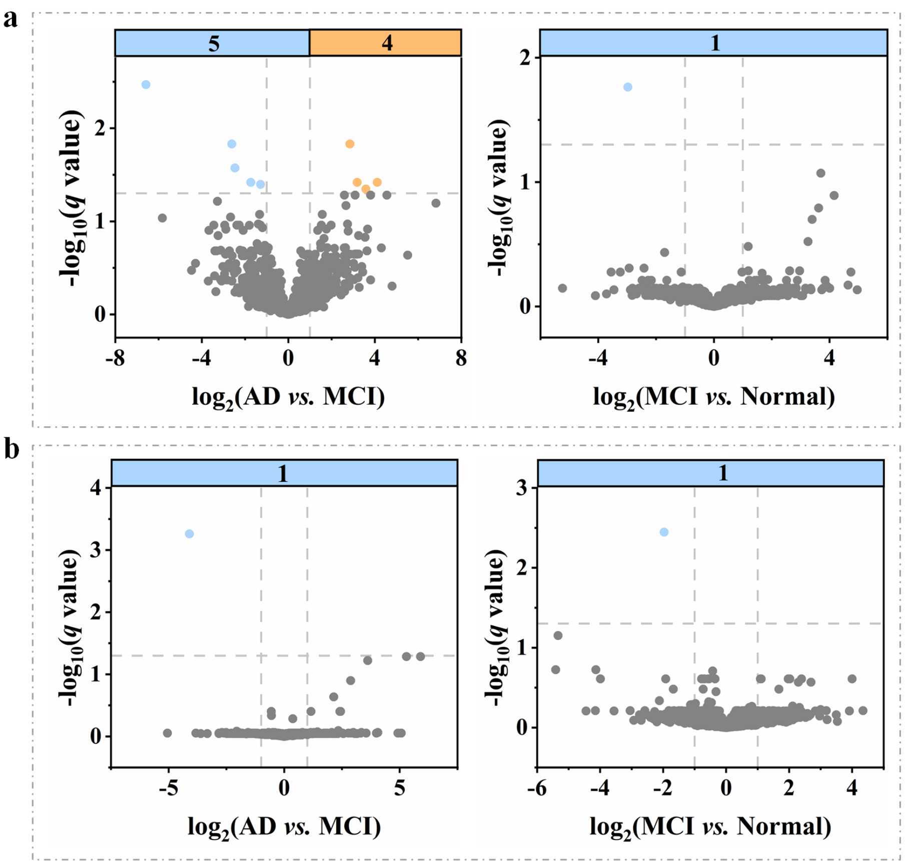

Figure S13. Volcano plots reveal dysregulated N-glycopeptides in serum (a) and CSF (b) based on pairwise comparisons among AD *vs.* MCI and MCI *vs.* Normal groups (|log2(fold change)| >1, *q* value < 0.05, with *q* values adjusted using the Benjamini-Hochberg correction). N-glycopeptides are shown in sky blue for downregulation and light orange for upregulation.

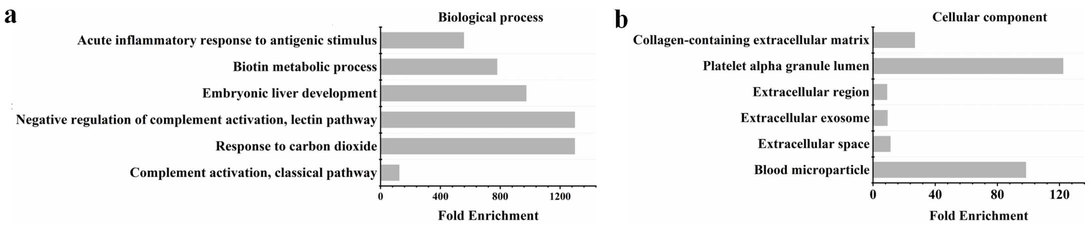

Figure S14. GO analysis of eight dysregulated glycoproteins shared between serum and CSF in the AD *vs.* Normal comparison. (a) Biological process and (b) cellular component categories.

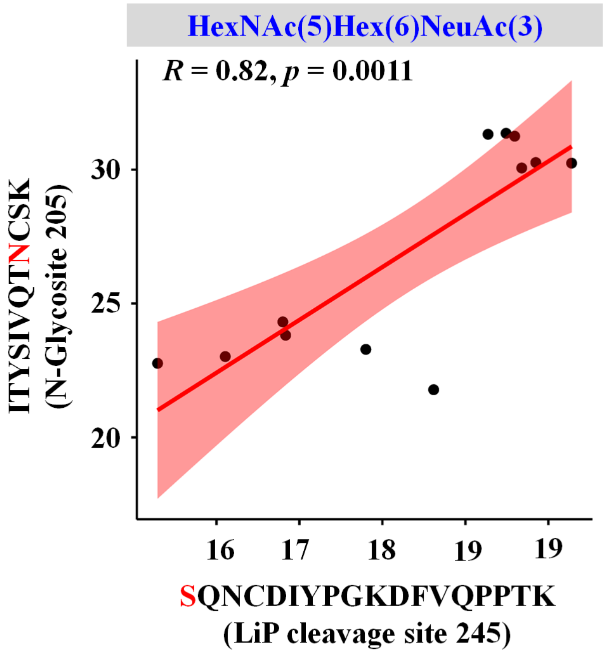

Figure S15. Correlation analysis between protein structural alterations and N-glycosylation in CSF kininogen-1 across pairwise comparisons. Pearson spearman correlation analyses were performed between an N-glycopeptide with the glycan composition HexNAc(5)Hex(6)NeuAc(3) and a LiP HT peptide. The X-axis represents the log_2-transformed intensity of the LiP HT peptide (with the LiP cleavage site highlighted in red), and the Y-axis represents the log_2-transformed intensity of the N-glycopeptide (with the N-glycosylation site highlighted in red).

Table S1. A summary of sample information, including age, gender, and disease conditions from the ADRC.

Sample	Participant Age	Gender	Disease conditions
adrc00030	67.01	M	Normal
adrc00081	68.26	M	Normal
adrc00108	68.89	M	Normal
adrc00111	71.06	F	Normal
adrc00115	72.39	M	Normal
adrc00196	66.07	M	Normal
adrc00003	80.37	F	MCI
adrc00095	81.43	F	MCI
adrc00219	79.63	F	MCI
adrc00341	81.39	M	MCI
adrc00359	84.41	F	MCI
adrc00360	81.03	M	MCI
adrc00001	81.82	M	AD
adrc00013	77.84	M	AD
adrc00024	83.72	M	AD
adrc00082	73.15	F	AD
adrc00214	80.61	F	AD
adrc00268	79.41	F	AD

(Note: M indicates male, F indicates female, MCI indicates mild cognitive impairment, and AD indicates Alzheimer's disease)
